# Supplementary material for: AAV-CRB2 protects against vision loss in an inducible CRB1 retinitis pigmentosa mouse model
Source: Mol Ther Methods Clin Dev. 2020 Dec 25;20:423–41. doi: 10.1016/j.omtm.2020.12.012 (PMC7848734; doi:10.1016/j.omtm.2020.12.012)
Supplement: Document S1. Figures S1–S5 [file mmc1.pdf]

OMTM, Volume *20*

Supplemental Information

AAV-*CRB2* protects against vision loss in an inducible *CRB1* retinitis pigmentosa mouse model

Thilo M. Buck, Rogier M. Vos, C. Henrique Alves, and Jan Wijnholds

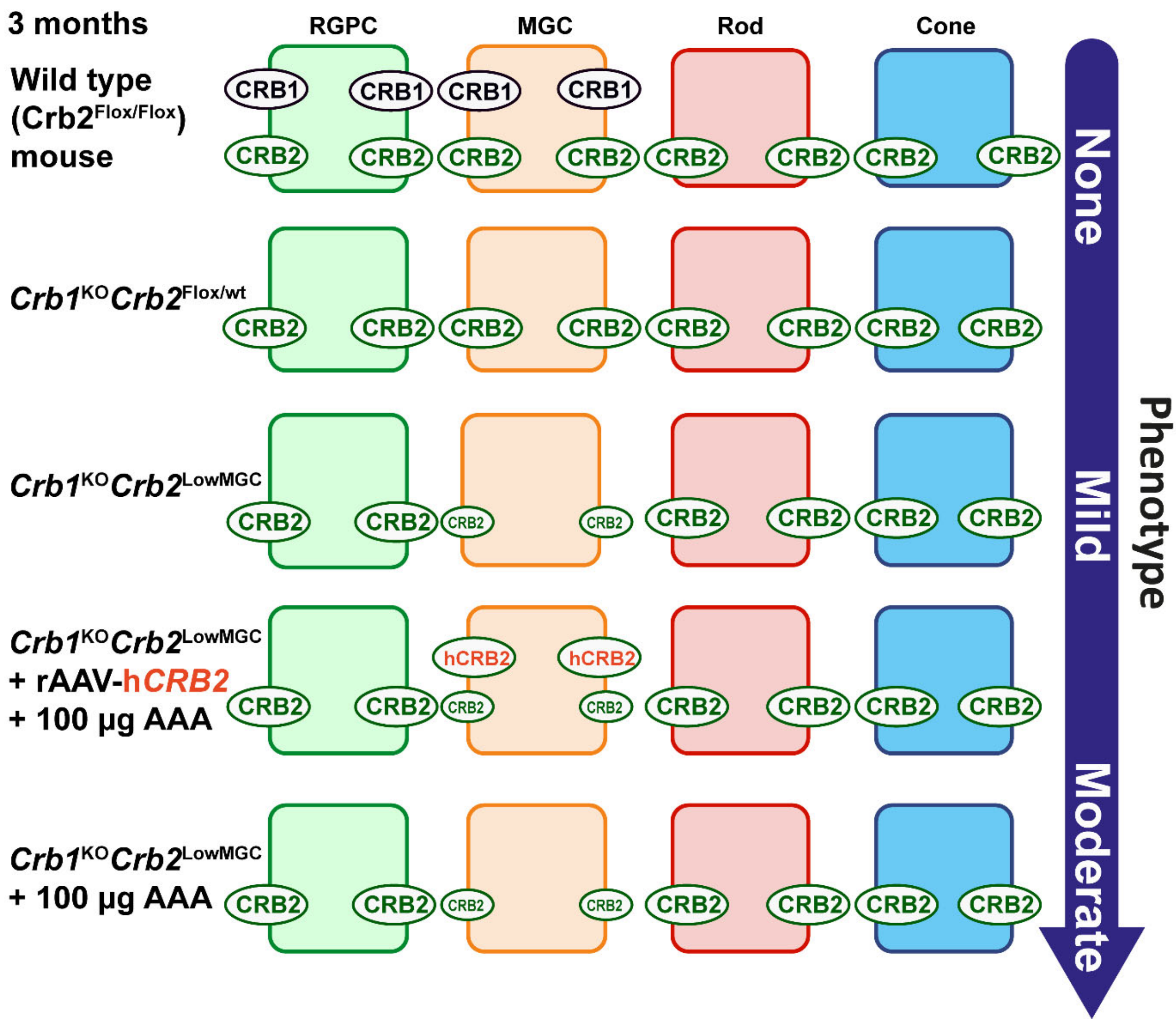

Figure S1. Graphical schematic representation of CRB localization and the severity of the phenotype in 3-month-old mice. Graphical schematic representation of CRB1 and CRB2 in radial glial progenitor cells (RGPC), Müller glial cells (MGC), rod and cone photoreceptors in wildtype (*Crb2*$^{\text{Flox/Flox}}$), *Crb1*^{KO}*Crb2*$^{\text{Flox/Flox}}$ (Control to *Crb1*^{KO}*Crb2*^{LowMGC}), *Crb1*^{KO}*Crb2*^{LowMGC}, *Crb1*^{KO}*Crb2*^{LowMGC} + 100 µg DL-AAA intravitreally (i.vit) injected at 2 months (Control to rAAV-*CRB2* injected mice), and *Crb1*^{KO}*Crb2*^{LowMGC} + rAAV-h*CRB2* i.vit injected at postnatal day 21 + 100 µg DL-AAA i.vit injected at 2 months. rAAV, recombinant adeno-associated viral vector; i.vit., intravitreal. Modified from [5].

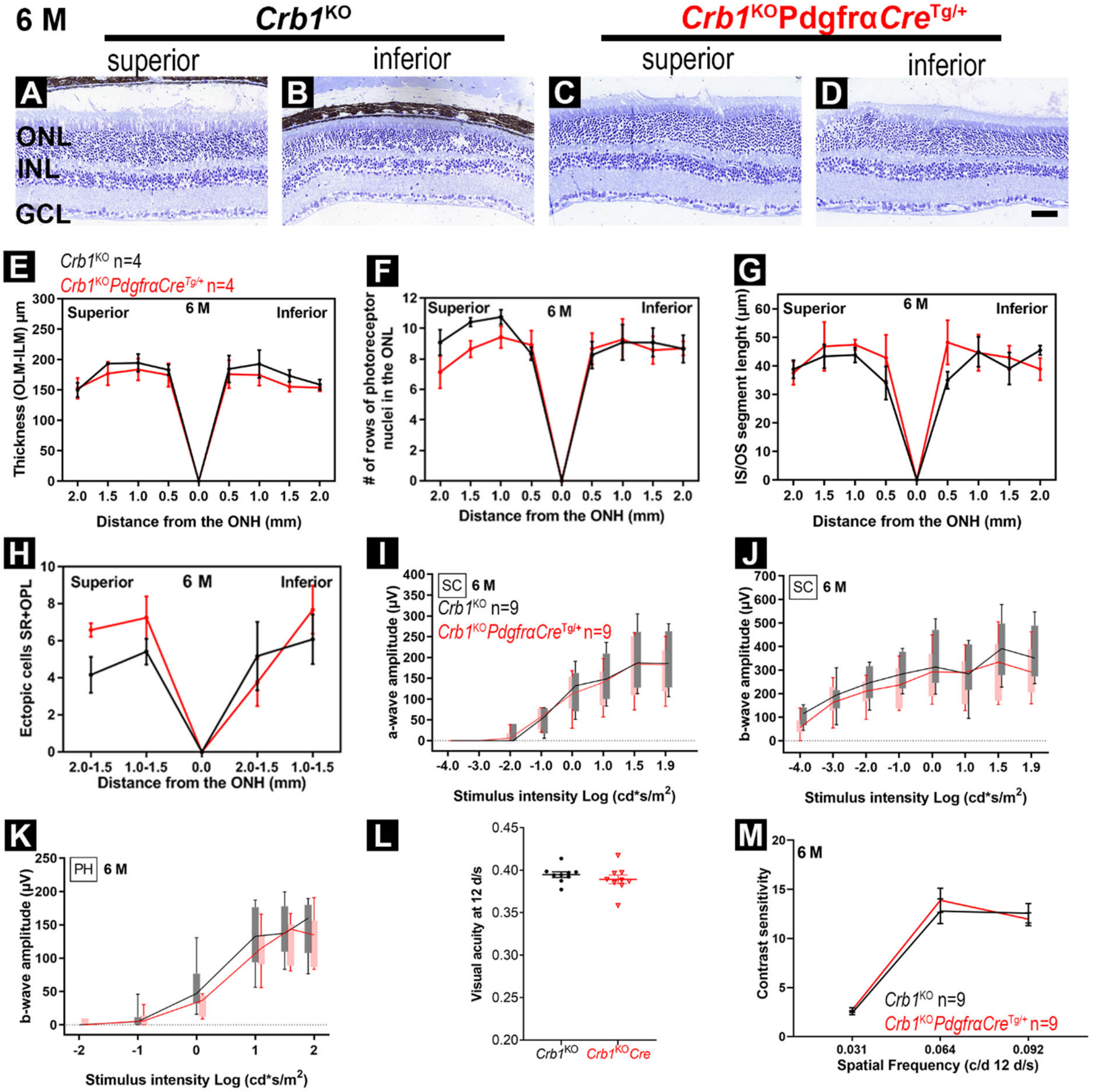

Figure S2. Cre-expression in Müller glial cells does not affect retinal morphology, ERG responses, and OKT responses. Toluidine-stained light microscopy of retinal sections from control (*Crb1*^{KO}; A-B) and *Crb1*$^{-/-}$ Pdgfrα*Cre*$^{Tg/+}$ (C-D) mice at 6-month-of-age. Spidergrams for (E) Retinal thickness, (F) number of photoreceptors per row, (G) inner/outer segments of photoreceptor length, and (H) ectopic cells in the subretinal space and outer plexiform layer. Quantitative evaluation of the scotopic single-flash intensity series of the a-wave (I), b-wave (J), and photopic b-wave (K) amplitudes in 6-month old mice. Boxes indicate the 25 and 75% quantile range, whiskers indicate the 5 and 95% quantiles, and the intersection of line and error bar indicates the median of the data (box-and-whisker plot). (L-M) Optokinetic head tracking response at 6-month old mice. (L) Spatial frequency threshold (visual acuity). (M) Contrast sensitivity threshold at different spatial frequencies. *P<0.05; **p<0.01, ***P<0.001.

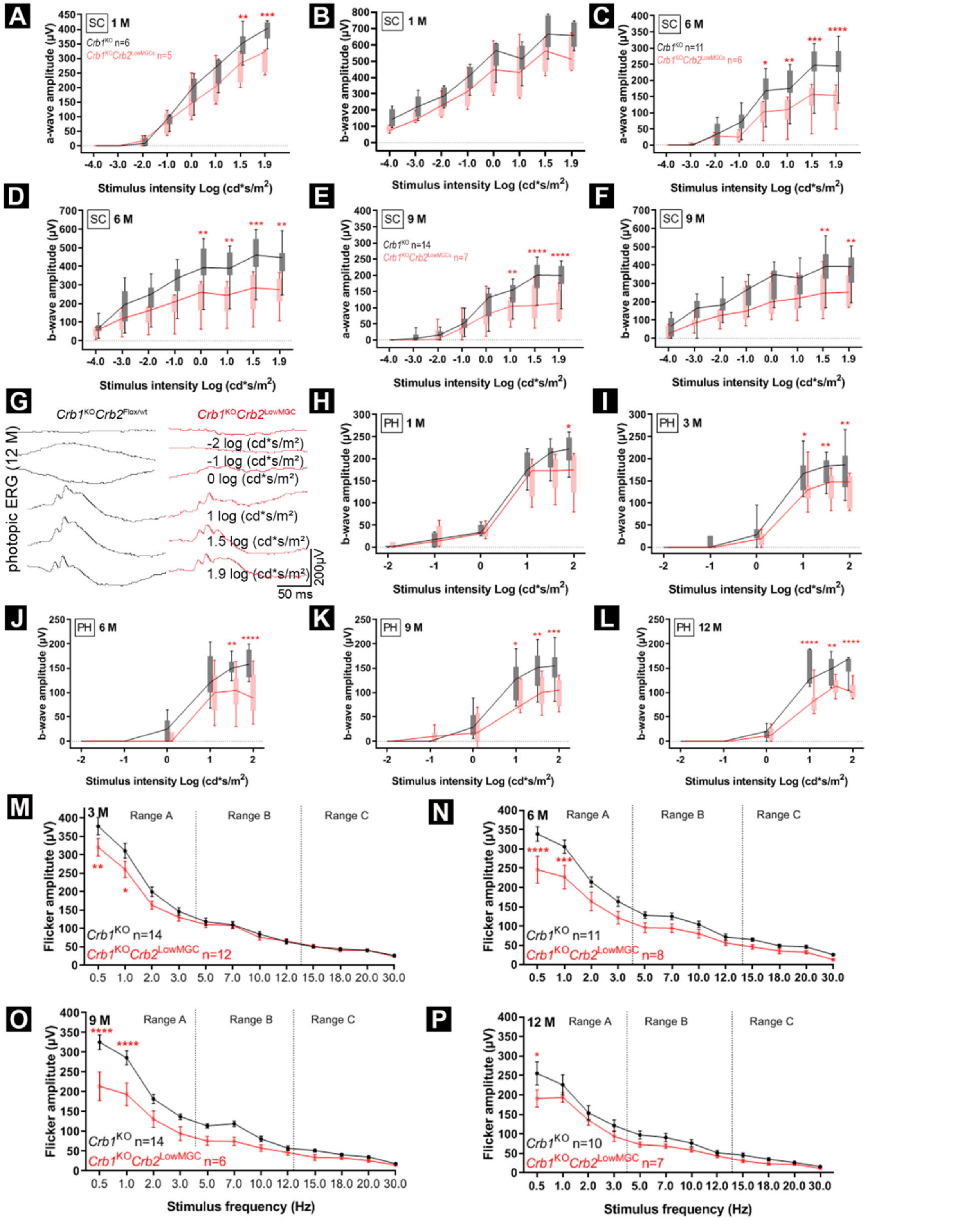

Figure S3. Decreased retinal function (ERG) and vision-guided (OKT) behavior in *Crb1*^{KO}*Crb2*^{LowMGC} compared to *Crb1*^{KO}*Crb2*$^{Flox/wt}$ age-matched littermates. *Crb1*^{KO}*Crb2*^{LowMGC} measurements are indicated in red (experimental group) and *Crb1*^{KO}*Crb2*$^{Flox/wt}$ age-matched littermates in black (control group). Electroretinographic analysis of the retinal function: (A-F) Scotopic [SC] single-flash intensity series ERG from representative animals at 1-, 6-, 9-month old mice of age. (G-L) Photopic [PH] single-flash ERG at different light intensities (-2, -1, 0, 1, 1.5, 1.9 log cd s/m² light intensity at 30 cd/m² background light): (G) Photopic ERG traces of representative 12-month-

old animals. (H-L) Photopic b-wave amplitudes at 1-, 3-, 6-, 9-, and 12-month-old mice (H-L). No statistical analysis was performed for (A-L). (M-P) Time course of flicker response amplitudes from 3-, 6-, 9-, and 12-month-old mice (Mean±SEM; M-P; *$P<0.05$; **$p<0.01$, ***$P<0.001$). Animal numbers (control vs. experimental): 1-month (n=6; n=6), 3-month (n=14; n=12), 6-month (n=11; n=6), 9-month (n=12; n=7), 12-month (n=7; n=8).

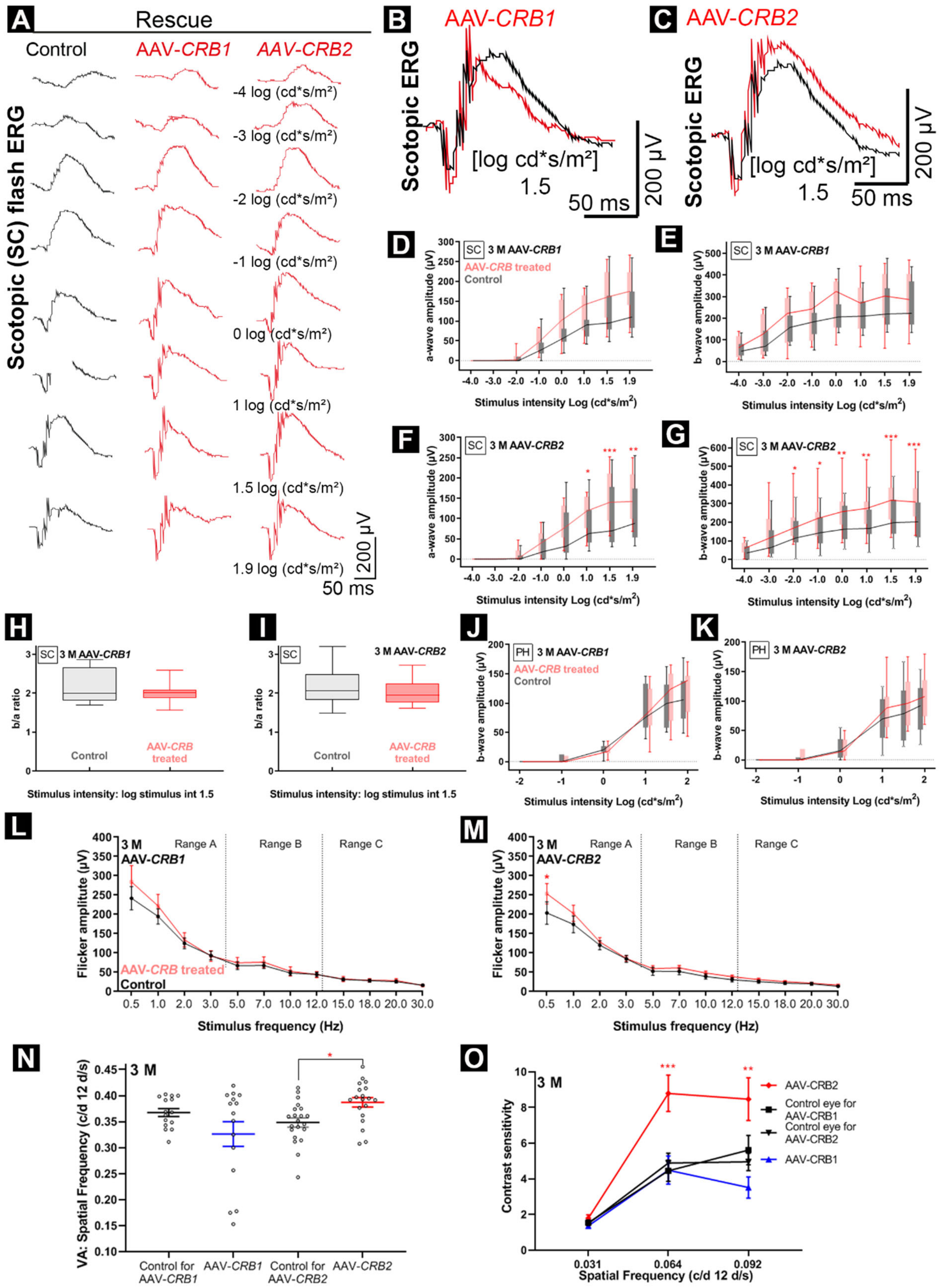

Figure S4. Retinal function and visual-guided head tracking in rAAV-h*CRB* treated *Crb1*[KO]*Crb2*[LowMGC] compared to control eyes. Both eyes received a 100 µg DL-AAA treatment at 2-months. Eyes were measured at three months. The ERG traces of the rAAV treated eye (rAAV-h*CRB* injected at postnatal day 21) are indicated in

red (experimental group) and compared to the control eye (Not treated) not receiving the AAV therapy (black trace, control group). Electroretinographic analysis of the retinal function: (A-C) Scotopic [SC] single-flash intensity series (-4, -3 , -2, -1, 0, 1, 1.5, 1.9 log cd s/m² light intensity) ERG from representative animals in 3-month old mice. (B-C) Superimposed scotopic [SC] single-flash ERG traces at 1.5 log cd s/m² intensity from representative animals. (D) Scotopic a-wave of rAAV-h*CRB1* injected mice vs. the control eye. (E) Scotopic b-wave of rAAV-h*CRB1* injected mice vs. control eye. (F) Scotopic a-wave of rAAV-h*CRB2* injected mice vs. control eye. (G) Scotopic b-wave of rAAV-h*CRB2* injected mice vs. control eye. (H-I) b-wave/a-wave ratio of AAV-h*CRB1* or AAV-h*CRB2* at single-flash ERG traces at 1.5 log cd s/m² intensity. (J-K) Photopic b-wave amplitudes in 3-month-old mice injected with rAAV-h*CRB1* or rAAV-h*CRB2* vs. control eye. No statistical analysis was performed (A-K). (L-M) Flicker response amplitudes from 3-month-old mice injected with rAAV-h*CRB1* or rAAV-h*CRB2* vs, the control eye. Number of animals (Control vs experimental): rAAV-h*CRB1* (n=16; n=14). rAAV-h*CRB2* (n=18; n=16). (N-P Optokinetic head tracking response: (N) Visual acuity (animals indicates as black circles); contrast sensitivity OKT (Number of animals: rAAV-h*CRB1* n=16; rAAV-h*CRB2*: n=21). Mean±SEM; M-P; *P<0.05; **p<0.01, ***P<0.001.

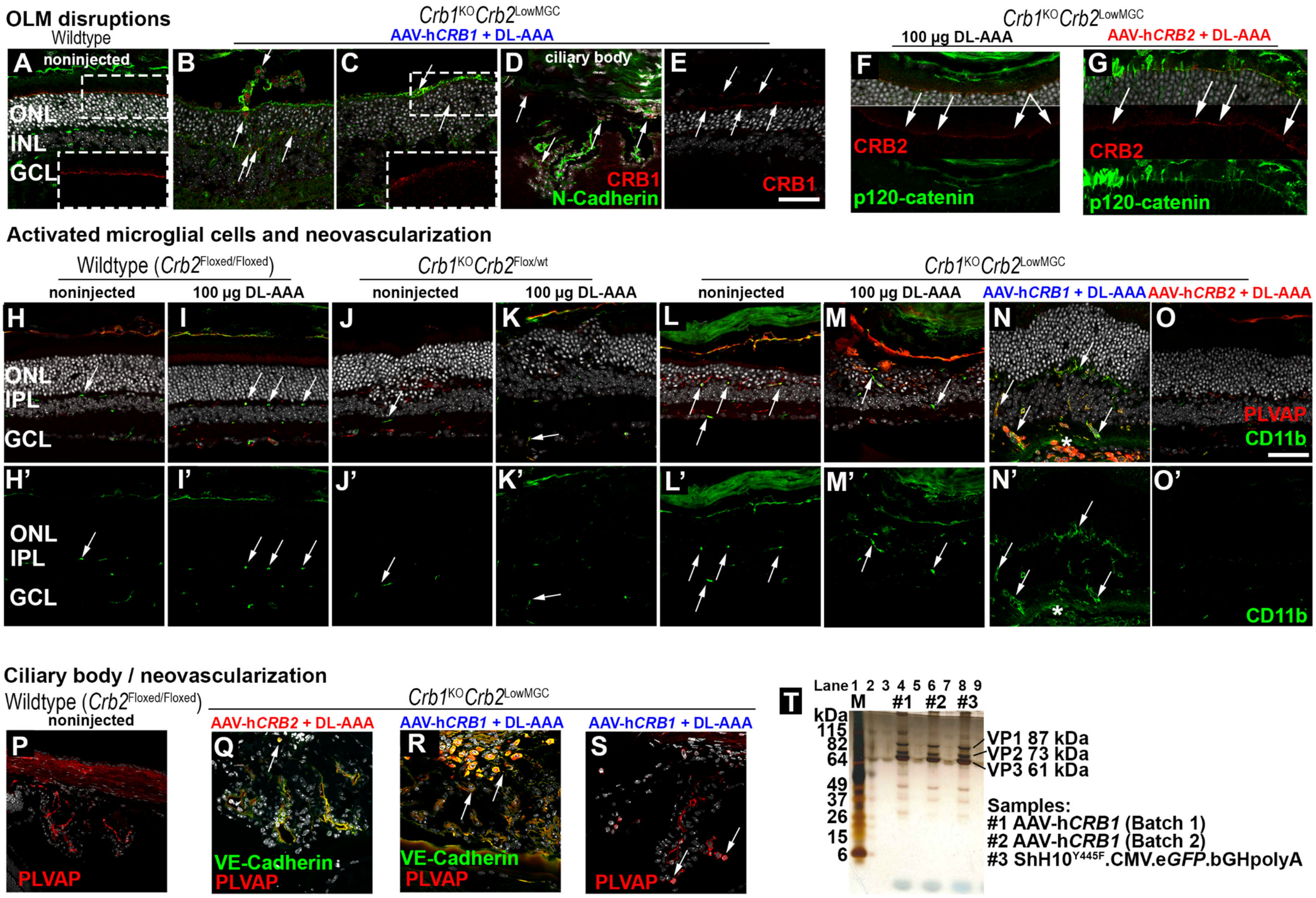

OLM disruptions
Wildtype
Crb1KO Crb2LowMGC
AAV-hCRB1 + DL-AAA
A noninjected
ONL
INL
GCL
B
C
D ciliary body
CRB1
N-Cadherin
E
CRB1
Crb1KO Crb2LowMGC
100 µg DL-AAA
AAV-hCRB2 + DL-AAA
F
G
CRB2
p120-catenin
Activated microglial cells and neovascularization
Wildtype (Crb2Floxed/Floxed)
Crb1KO Crb2Flox/wt
Crb1KO Crb2LowMGC
noninjected
100 µg DL-AAA
AAV-hCRB1 + DL-AAA
AAV-hCRB2 + DL-AAA
H I J K L M N O
ONL
IPL
GCL
PLVAP
CD11b
H' I' J' K' L' M' N' O'
Ciliary body / neovascularization
Wildtype (Crb2Floxed/Floxed)
noninjected
P
PLVAP
Q
R
S
VE-Cadherin
T
Lane 1 2 3 4 5 6 7 8 9
M #1 #2 #3
kDa
115
82
64
49
37
26
15
6
VP1 87 kDa
VP2 73 kDa
VP3 61 kDa
Samples:
#1 AAV-hCRB1 (Batch 1)
#2 AAV-hCRB1 (Batch 2)
#3 ShH10Y445F.CMV.eGFP.bGHpolyA

Figure S5. AAV-h*CRB1* increases neovascularization in the ciliary body. Immunohistochemistry of 3-month-old mice. Sections were stained for: (A-E) CRB1 protein (red) at the subapical region and N-cadherin (green) at the adherens junction in (A) wildtype and (B-E) *Crb1*^{KO}*Crb2*^{LowMGC} mice injected with rAAV-h*CRB1* and 100 µg DL-AAA (arrows indicate CRB1 protein expression). (F-G) CRB2 protein (red) at the subapical region and p120-catenin (green) at the adherens junction in (F) *Crb1*^{KO}*Crb2*^{LowMGC} mice injected with 100 µg DL-AAA and (G) *Crb1*^{KO}*Crb2*^{LowMGC} mice injected with 100 µg DL-AAA and rAAV-h*CRB2* (arrows = CRB2 protein expression at OLM). (H-O) PLVAP (red) for (neo-)vascularization and CD11b (green) for microglial activation (arrows). (H'-O') Puncta-like CD11b-positive microglia cells in IPL are dormant and activated CD11b-positive microglia cells in ONL and GCL (arrows). (P-S) The ciliary body of (P) wildtype control mice or *Crb1*^{KO}*Crb2*^{LowMGC} mice injected with (Q) rAAV-h*CRB2* or (R-S) rAAV-h*CRB1*. PLVAP (red) and VE-cadherin (green) are vascularization markers. (Neo-)vascularization indicated by arrows in Q-S. (T) $1x10^{10}$ viral genomes (vg) of two batches of rAAV2/ShH10^{Y445F}.CMVmin.h*CRB1* vector preparation and one batch of $1x10^{10}$ vg of rAAV2/ShH10^{Y445F}.CMV.*GFP* were analysed by silver nitrate staining to detect capsid proteins VP1, VP2, VP3. Note that the silver stained SDS-PAGE gel was overstained (for marker and capsid proteins) to allow evaluation of capsid degradation products and contaminants. Lane 1, protein marker. Lanes 4 and 6, two independent samples of rAAV2/ShH10^{Y445F}.h*CRB1* used in this study. Lane 8, a sample of rAAV2/ShH10^{Y445F}.*GFP*. Note that lanes 2, 3, 5, 7 and 9 contain overflow from adjacent sample wells. Scale bar=50 µm. Inserts 50 µm height. PLVAP, plasmalemma vesicle associated protein; ONL, outer nuclear layer; INL, inner nuclear layer; GCL, ganglion cell layer. 3-4 eyes per group were analyzed.
