# Supplementary material for: AAV-CRB2 protects against vision loss in an inducible CRB1 retinitis pigmentosa mouse model
Source: Mol Ther Methods Clin Dev. 2020 Dec 25;20:423–41. doi: 10.1016/j.omtm.2020.12.012 (PMC7848734; doi:10.1016/j.omtm.2020.12.012)
Supplement: Document S2. Article plus supplemental information [file mmc2.pdf]

Molecular Therapy
Methods & Clinical Development
Original Article

AAV-*CRB2* protects against vision loss in an inducible *CRB1* retinitis pigmentosa mouse model

Thilo M. Buck,[1] Rogier M. Vos,[2] C. Henrique Alves,[1,3] and Jan Wijnholds[1,2]

[1]Department of Ophthalmology, Leiden University Medical Center (LUMC), 2333 ZC Leiden, the Netherlands; [2]Netherlands Institute of Neuroscience, Royal Netherlands Academy of Arts and Sciences (KNAW), 1105 BA Amsterdam, the Netherlands

Loss of Crumbs homolog 1 (CRB1) or CRB2 proteins in Müller cells or photoreceptors in the mouse retina results in a CRB dose-dependent retinal phenotype. In this study, we present a novel Müller cell-specific *Crb1*^{KO}*Crb2*^{LowMGC} retinitis pigmentosa mouse model (complete loss of CRB1 and reduced levels of CRB2 specifically in Müller cells). The *Crb* double mutant mice showed deficits in electroretinography, optokinetic head tracking, and retinal morphology. Exposure of retinas to low levels of DL-α-aminoadipate acid induced gliosis and retinal disorganization in *Crb1*^{KO}*Crb2*^{LowMGC} retinas but not in wild-type or *Crb1*-deficient retinas. *Crb1*^{KO}*Crb2*^{LowMGC} mice showed a substantial decrease in inner/outer photoreceptor segment length and optokinetic head-tracking response. Intravitreal application of rAAV vectors expressing human *CRB2* (h*CRB2*) in Müller cells of *Crb1*^{KO}*Crb2*^{LowMGC} mice subsequently exposed to low levels of DL-α-aminoadipate acid prevented loss of vision, whereas recombinant adeno-associated viral (rAAV) vectors expressing human *CRB1* (h*CRB1*) did not. Both rAAV vectors partially protected the morphology of the retina. The results suggest that h*CRB* expression in Müller cells is vital for control of retinal cell adhesion at the outer limiting membrane, and that the rAAV-cytomegalovirus (CMV)-h*CRB2* vector is more potent than rAAV-minimal CMV (CMVmin)-h*CRB1* in protection against loss of vision.

INTRODUCTION

Mutations in the Crumbs homolog 1 (*CRB1*) gene are associated with retinitis pigmentosa (RP), Leber congenital amaurosis (LCA), and cone-rod dystrophies and are sporadically found in foveal retinoschisis and macular dystrophy.[1–3] The human and nonhuman primate retina express and localize CRB1 and CRB2 proteins in Müller glial cells (MGCs) and photoreceptor cells (PRCs) at the outer limiting membrane (OLM).[4–6] The mouse retina also expresses and localizes the CRB2 protein at the OLM in MGCs and PRCs. However, whereas the mouse retina does express and localize the CRB1 protein at the OLM in MGCs and retinal progenitor cells, the mouse retina does not express the CRB1 protein in PRCs. The human and nonhuman primate retinas express a CRB1 protein of 1,406 aa, whereas the mouse retina expresses a CRB1 protein of 1,405 aa.[7–9] Loss of the CRB1 or the CRB2 protein in the retina results in loss of adhesion between MGCs, between PRCs, and among MGCs and PRCs.[5,8,10–12] No therapy is available for the treatment of *CRB1*-related retinal dystrophies. Recombinant adeno-associated viral (rAAV) vector-mediated gene supplementation may provide a lasting therapy to *CRB1* RP patients. We previously showed that CRB2 can rescue retinas lacking CRB1 or CRB2 proteins from retinal degeneration in two fast-progression RP mouse models by increasing the levels of CRB2 into both MGCs and PRCs.[13] However, rescue at mid-stage retinal disease could not be achieved with *CRB1* cDNA supplementation, or by supplementation of *CRB2* cDNA only in PRCs or only in MGCs. In the present study, we developed a sensitive RP MGC-specific mouse model to test for protection at early stage retinal disease by rAAV-human *CRB* (h*CRB*) gene therapy vectors.

We also investigated the development of the mouse retinal phenotype to explore the window of opportunity for rAAV-h*CRB* gene therapy. Previously, we analyzed *Crb*-related retinal degeneration mouse models. (1) The knockout (KO) of the *Crb1* gene (*Crb1*^{KO}) ablates the expression of the CRB1 protein in MGCs and retinal precursor cells, and it resulted in slow progression of retinal disorganization and degeneration from postnatal day 14 (P14) on.[8,12,14] (2) In *Crb1*^{KO} mice, retinal degeneration occurs at foci in the inferior temporal quadrant of the retina. (3) Cell-type specific ablation of CRB1 in MGCs, or of CRB2 in MGCs, suggested that CRB proteins execute important overlapping roles in MGCs. Loss of CRB1 protein in mouse MGCs, or the loss of CRB2 in MGCs, results in disruptions at the OLM, protrusion of rows of photoreceptor nuclei into the photoreceptor inner and outer segment layers, and ingressions of rows of photoreceptor nuclei into the outer plexiform layer (OPL). These retinas mimic RP in which the retinal degeneration process remains slow during the period of 1 year.[5,6,11,15,16] (4) Most importantly, the complete loss of CRB2 in MGCs in *Crb1*^{KO} mice worsened the retinal

Received 11 August 2020; accepted 21 December 2020;
https://doi.org/10.1016/j.omtm.2020.12.012.

[3]Present address: Coimbra Institute for Clinical and Biomedical Research (iCBR), Faculty of Medicine, University of Coimbra, 3000-548 Coimbra, Portugal.

Correspondence: Jan Wijnholds, Department of Ophthalmology, Leiden University Medical Center (LUMC), 2333 ZC Leiden, the Netherlands.
E-mail: j.wijnholds@lumc.nl

This is an open access article under the CC BY license (http://creativecommons.org/licenses/by/4.0/).

phenotype from a RP-like to LCA-like phenotype.[5,9,16] These *Crb1*^{KO}*Crb2*$^{\Delta MGC}$ retinas lacking CRB1 and CRB2 specifically in MGCs show in addition to protrusion of photoreceptor nuclei into the segment layers, also an intermingling of photoreceptors with inner retinal cells. The *CRB1* RP and *CRB2* MGC-specific RP models are not suitable for testing gene therapy vectors since the onset of retinal degeneration is too slow, whereas the *Crb1*^{KO}*Crb2*$^{\Delta MGC}$ MGC-KO LCA model is not suitable to test gene therapy vectors because the onset of retinal degeneration occurs during retinal development and is too fast. In this study, we analyzed a novel mouse model by reducing endogenous mouse CRB2 (mCRB2) expression in MGCs from one instead of two *Crb2* alleles in *Crb1*^{KO} mice (*Crb1*^{KO}*Crb2*^{LowMGC}). Compared to littermate control *Crb1*^{KO} *Crb2*$^{Flox/WT}$ (floxed/wild-type *Crb2*) the *Crb1*^{KO}*Crb2*^{LowMGC} showed a worsened retinal phenotype; therefore, we used these mice to test rAAV human *CRB1* (h*CRB1*) and human *CRB2* (h*CRB2*) gene therapy vectors that specifically target the MGCs.

Many underlying diseases show a nominal phenotype until a stressor triggers an escalation. DL-α-aminoadipate acid (DL-AAA)-mediated MGC-specific stress causes disruptions at the OLM and protrusion of photoreceptor nuclei into the segment layers.[17] DL-AAA is a cystine/glutamate-specific antiporter inhibitor, decreasing the reserve pool of cysteine and glutathione (GSH) in MGCs.[18] Low doses of DL-AAA disrupt the distal Müller glial sealing at the OLM by downregulation of the adherens junction-associated protein zonula occludens-1 (ZO-1).[19] The decrease in adhesion mediated by DL-AAA intravitreal injection is linked to photoreceptor nuclei protrusions in control mice and in the S334ter-line-3 rat model of RP.[17,19] First, a decrease of glial fibrillary acidic protein (GFAP) was found 3 days after intravitreal injection of DL-AAA in the S334ter-line-3 rat model of RP, followed by an upregulation of GFAP 2 weeks later.[19] Subretinal injection of DL-AAA to nonhuman primates caused a reduction in photoreceptor nuclei and a decrease in the electroretinography (ERG) response.[20] The long-term effects of low doses of DL-AAA on vision-guided behavior in RP models have not been thoroughly investigated.

Here, we studied the effects of DL-AAA on retinas with decreased levels of CRB2 in MGCs lacking CRB1 (*Crb1*^{KO}*Crb2*^{LowMGC}) compared to retinas with normal levels of CRB2 in MGCs lacking CRB1 (*Crb1*^{KO}*Crb2*$^{Flox/WT}$). We challenged the following mice on a 99.9% C57BL/6JOlaHsd genetic background to DL-AAA: *Crb2*$^{Flox/Flox}$ control mice that do not express Cre recombinase, and two RP mouse models (*Crb1*^{KO}*Crb2*$^{Flox/WT}$ mice not expressing Cre recombinase, and *Crb1*^{KO}*Crb2*^{LowMGC} mice that express Cre recombinase specifically in MGCs to ablate one allele of *Crb2*). Our data suggest that *Crb1*^{KO}*Crb2*$^{Flox/WT}$ and *Crb2*$^{Flox/Flox}$ retinas are less sensitive to DL-AAA than are *Crb1*^{KO}*Crb2*^{LowMGC} retinas, suggesting that raising the levels of CRB2 by rAAV gene therapy targeting *Crb1*^{KO}*Crb2*^{LowMGC} MGCs might prevent the adverse effects of the glial toxin DL-AAA.

In summary, we demonstrate that (1) low levels of CRB2 in *Crb1*^{KO}*Crb2*^{LowMGC} MGCs lacking CRB1, with normal levels of CRB2 expressed at the OLM in photoreceptors (see the cartoon in Figure S1 on CRB protein expression in the mouse model), result in a RP retinal phenotype with foci of retinal disorganization mostly in the inferior retina. Interestingly, our previous studies showed that complete loss of CRB2 in MGCs lacking CRB1 resulted in an LCA retinal phenotype throughout the entire retina.[5] Furthermore, we show that (2) reduction of CRB2 protein levels worsens the retinal phenotype in the inferior quadrants as found in *Crb1*^{KO} mice, (3) *Crb1*^{KO}*Crb2*^{LowMGC} RP mice are more susceptible to stress on MGCs than are *Crb1*^{KO}*Crb2*$^{Flox/WT}$ and *Crb2*$^{Flox/Flox}$ mice, and (4) rAAV-h*CRB2* therapy protects *Crb1*^{KO}*Crb2*^{LowMGC} retinas against loss of vision due to exposure to DL-AAA.

RESULTS

Reduction of CRB2 and loss of CRB1 in MGCs leads to ERG and OKT deficits

We expressed *Cre* under control of the Pdgfrα promoter in MGCs to ablate *Crb2* expression of one floxed allele (*Crb1*^{KO}*Crb2*^{LowMGC} = *Crb1*$^{-/-}$*Crb2*$^{Flox/WT}$ Pdgfrα*Cre*$^{Tg/+}$; Figure S1). High levels of Cre recombinase expression in photoreceptors and other neuronal cells can cause toxicity, impairing neuronal function.[21,22] We assessed whether *Cre* expression in *Crb1*^{KO} MGCs (*Crb1*$^{-/-}$Pdgfrα*Cre*$^{Tg/+}$) has an impact on retinal morphology, retinal transmission (ERG responses), or vision-guided optokinetic head-tracking thresholds (optokinetic tracking [OKT] response). No adverse effects were found (Figure S2).

Next, we measured flash ERGs and OKT responses in 1-, 3-, 6-, 9-, and 12-month-old *Crb1*^{KO}*Crb2*^{LowMGC} mice and age-matched littermate controls (*Crb1*^{KO}*Crb2*$^{Flox/WT}$. Figures 1A–1J; Figure S3). One-month-old *Crb1*^{KO}*Crb2*^{LowMGC} mice showed normal ERG responses (scotopic, photopic, flicker) and OKT thresholds (visual acuity [VA] thresholds; contrast sensitivity thresholds) compared to age-matched littermate controls (Figures S3A, S3B, and S3H). Three-month-old dark-adapted *Crb1*^{KO}*Crb2*^{LowMGC} mice showed a reduced a-wave and b-wave response (Figures 1A–1D) and a reduced ERG flicker response (0.5 Hz; Figure S3M) compared to *Crb1*^{KO}*Crb2*$^{Flox/WT}$ mice, suggesting a reduced rod photoreceptor retinal function. The ERG dark-adapted a-wave and b-wave response worsened over time in *Crb1*^{KO}*Crb2*^{LowMGC} and *Crb1*^{KO}*Crb2*$^{Flox/WT}$ mice (6-, 9-, 12-month-old mice; Figures 1A and 1E–1G; Figure S3). The a-wave and b-wave amplitudes were proportionally lower (Figures 1H and 1I), but the b-wave/a-wave ratio of the scotopic ERG was not affected (Figure 1J), indicating that the overall retinal transmission (b-wave) and the photoreceptor response (a-wave) were impeded.

Vision-guided OKT responses (Figure 1K) were assessed on visual acuity (spatial frequency) and contrast sensitivity measurements.[23–25] The spatial frequency threshold (visual acuity) was lower in 12-month-old *Crb1*^{KO}*Crb2*^{LowMGC} mice compared to *Crb1*^{KO}*Crb2*$^{Flox/WT}$ littermates (Figure 1L). The contrast sensitivity threshold was markedly lower already at 6 and 12 months at a wide range of spatial frequencies measured (Figure 1M). The effects on contrast sensitivity threshold differences were detected with a higher statistical significance level at the spatial frequencies 0.064 and 0.092 cycles/degree (c/d; Figure 1M),

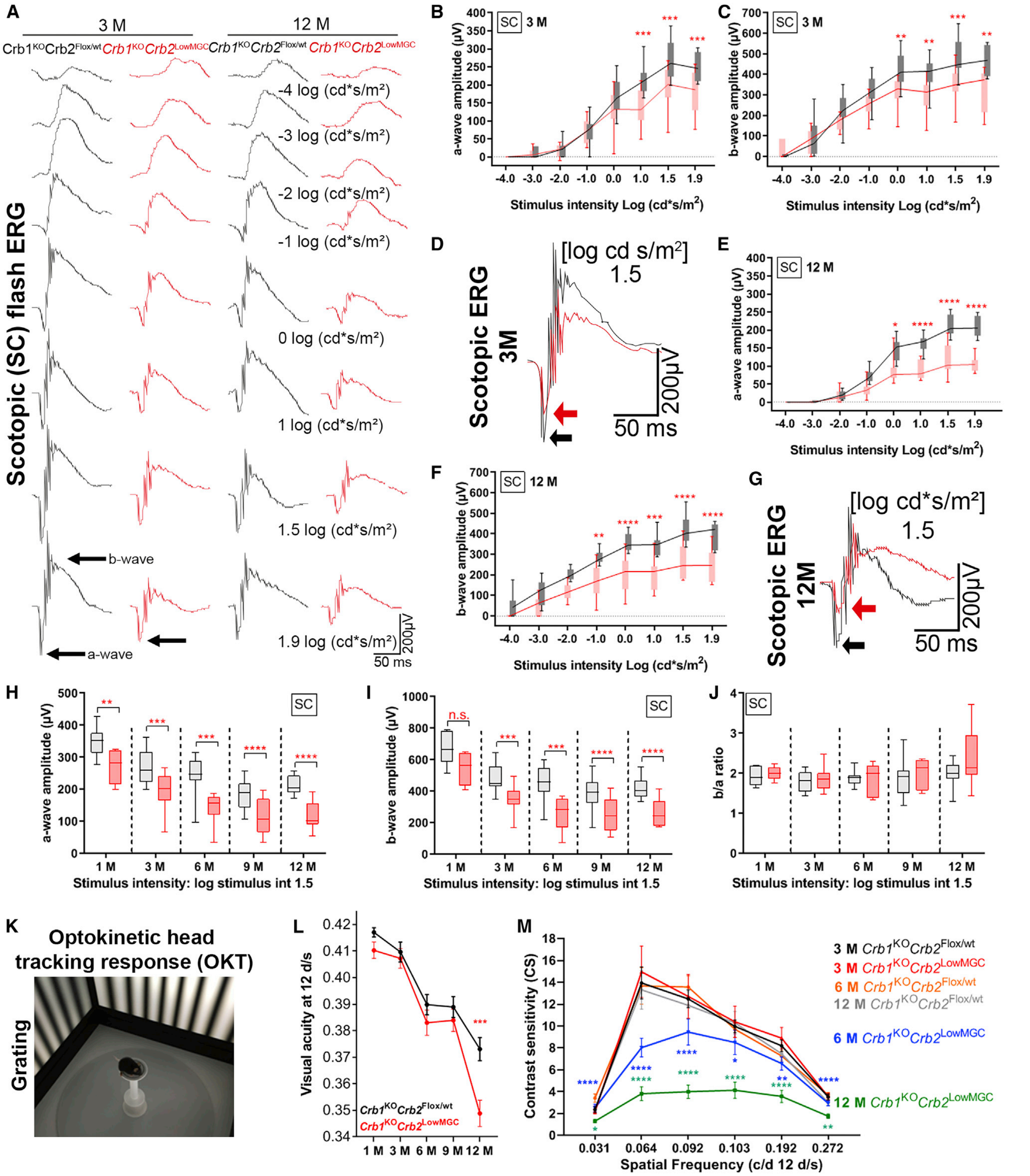

Figure 1. Decreased retinal function and vision-guided behavior in *Crb1*^{KO}*Crb2*^{LowMGC} compared to *Crb1*^{KO}*Crb2*$^{Flox/WT}$ age-matched littermates

Crb1^{KO}*Crb2*^{LowMGC} measurements are indicated in red (experimental group), and *Crb1*^{KO}*Crb2*$^{Flox/WT}$ age-matched littermates with a similar genetic background are indicated in black (control group). (A) Electroretinographic analysis of retinal function: scotopic single-flash intensity series (−4, −3, −2, −1, 0, 1, 1.5, and 1.9 log cd·s/m^2 light

(legend continued on next page)

suggesting that these spatial frequencies are most informative for h*CRB* gene therapy studies.

Reduction of CRB2 in MGCs results in a more severe CRB1 phenotype in the inferior part of the retina

We analyzed the morphological phenotype on mouse eyes on plastic sections. We included a WT-like mouse with a similar genetic background (*Crb2*$^{Flox/Flox}$) because ectopic cell counts and photoreceptor inner/outer segment (IS/OS) length quantification compared to *Crb1*^{KO}*Crb2*$^{Flox/WT}$ mice on plastic sections had not been done previously on mice with a 99.9% C57BL/6JOlaHsD genetic background. At 3 months of age, in *Crb1*^{KO}*Crb2*^{LowMGC} retinas compared to littermate control *Crb1*^{KO}*Crb2*$^{Flox/WT}$ retinas, disorganization of the retinal layering at foci was detected in the two inferior quadrants of the retina (Figures 2A–2E; symbols: arrows, protrusions; open triangles, loss of photoreceptor IS/OS; asterisks, neovascularization). These disorganizations at foci included protrusions of photoreceptor nuclei into the photoreceptor segment layers, ingression of photoreceptor nuclei into the OPL, disruptions at the OLM, and intermingling PRCs with inner retinal cells. Interestingly, outside the foci of retinal disorganization the layering of the retina remained intact. At 12 months of age the severity and number of retinal disorganizations at foci increased. Retinal disorganization could also be observed in the littermate control *Crb1*^{KO}*Crb2*$^{Flox/WT}$ retinas, but outside of the affected foci the retinal layering remained intact (Figures 2F–2J). In the superior retina of the *Crb1*^{KO}*Crb2*^{LowMGC} mice, but not of the *Crb1*^{KO}*Crb2*$^{Flox/WT}$ mice, at 3 months of age, sporadic protrusions of photoreceptor nuclei at foci could also be detected (Figure 2D), and such protrusions were observed at 12 months of age in *Crb1*^{KO}*Crb2*^{LowMGC} retinas as well as in littermate control *Crb1*^{KO}*Crb2*$^{Flox/WT}$ retinas (Figures 2I and 2G). At 12 months of age, we observed that four out of five *Crb1*^{KO}*Crb2*^{LowMGC} retinas, and one out of five in the littermate *Crb1*^{KO}*Crb2*$^{Flox/WT}$ control retinas, developed focal neovascularization in the inferior quadrants (Figure 2J, asterisks).

We generated retinal spidergrams for retinal thickness, outer nuclear layer (ONL) thickness, inner nuclear layer (INL) thickness, number of rows of photoreceptor nuclei in the ONL, photoreceptor IS/OS length, ectopic cells in the subretinal space, and ectopic cells in the OPL. In 12-month-old mice, the retinal thickness, the number of rows of photoreceptor nuclei in the ONL, and the ONL thickness were decreased compared to *Crb1*^{KO}*Crb2*$^{Flox/WT}$ mice (Figures 2L, 2M, and 2O). No major difference was found in the INL thickness in 12-month-old mice (Figure 1N). *Crb1*^{KO}*Crb2*^{LowMGC} mice displayed many displaced retinal cells (at 1, 3, and 6 months of age; Figures 2E and 2P–2R). Most of the ectopic nuclei were found in the OPL and some at the subretinal space (compare Figure 2S and Figure 2Q). The photoreceptor IS/OS length in the inferior quadrants of *Crb1*^{KO}*Crb2*^{LowMGC} retinas was shorter compared to *Crb1*^{KO}*Crb2*$^{Flox/WT}$ retinas at 3 months, and both mouse lines had similar but shorter IS/OS in the inferior quadrants compared to the superior quadrants at 6 months of age (Figures 2T and 2U).

We also assessed the morphology on protein expression by immunohistochemistry in 3-month-old mice for gliosis, Müller glial microvilli, IS/OS of photoreceptors, synapses at the OPL and IPL, and OLM disruptions (Figure 3). More GFAP-positive stress fibers extending from the inner limiting membrane (ILM) to the OLM were observed in the inferior and superior quadrants of *Crb1*^{KO}*Crb2*^{LowMGC} compared to *Crb1*^{KO}*Crb2*$^{Flox/WT}$ mouse littermate retinas (Figures 3A–3E). When further looking at Müller glial morphology, also shortened and collapsed microvilli ($CD44^+$) were observed in *Crb1*^{KO}*Crb2*^{LowMGC} compared to *Crb1*^{KO}*Crb2*$^{Flox/WT}$ retinas (Figures 3F–3J). This matches our previous transmission electron microscopy (TEM) observation that the CRB levels from photoreceptors at the OLM and here in MGCs are important for MGC microvilli extensions.[11,12] Also, the outer segments of photoreceptors (labeled with cone arrestin for cones and rhodopsin for rods) were lost at foci in the inferior and partially in the superior quadrants of *Crb1*^{KO}*Crb2*^{LowMGC} compared to *Crb1*^{KO}*Crb2*$^{Flox/WT}$ retinas (Figures 3K–3O). Similarly, the internalization of rhodopsin around the cell nucleus was found in photoreceptor ingressions (Figures 3L, 3M, and 3O, asterisks). The IPL synapses (sublamina-a OFF-bipolar cell synapses; sublamina-b ON-bipolar cell synapses) were relatively unaffected as shown by the synaptic marker plasma membrane calcium ATPase 1 (PMCA1), but the OPL synapses (protein kinase Cα [PKCα]/PMCA1 markers) were disrupted in the superior and inferior quadrants of the retina of *Crb1*^{KO}*Crb2*^{LowMGC} compared to *Crb1*^{KO}*Crb2*$^{Flox/WT}$ retinas (Figures 3P–3T, asterisks indicate OPL disruption).[27] We also found many more OLM disruptions and a lower CRB2 expression at the OLM in *Crb1*^{KO}*Crb2*^{LowMGC} compared to *Crb1*^{KO}*Crb2*$^{Flox/WT}$ and WT-like (Crb2$^{Flox/Flox}$) retinas (Figures 3U–3Y, asterisks indicate OLM disruptions).

We previously hypothesized that the loss of mCRB2 protein expression at the OLM determines the retinal phenotype in *Crb1*^{KO} mice,

intensity) ERG from representative animals at 3 months (n = 14 control group; n = 12 experimental group) and 12 months of age (n = 8 per group). (B, C, E, F, H, and I) Quantitative evaluation of the scotopic single-flash ERG intensity series of the a-wave (B, E, and H) and b-wave (C, F, and I). (D–G) Superimposed scotopic single-flash ERG traces at 1.5 log cd·s/m^2 intensity from representative animals at 3 and 12 months of age. (H–J) Quantitative evaluation of the scotopic a-wave, b-wave, and b-wave/a-wave ratio at 1.5 log cd·s/m^2 intensity. Boxes indicate the 25% and 75% quantile range, whiskers indicate the 5% and 95% quantiles, and the intersection of the line and error bar indicates the median of the data (box-and-whisker plot). (K–M) Optokinetic head-tracking response for 1-, 3-, 6-, and 12-month-old mice (mean ± SEM). (L) Spatial frequency threshold (visual acuity) (number of animals [control versus experimental]): 1 month old (n = 13, n = 17), 3 months old (n = 23, n = 20), 6 months old (n = 23, n = 16), 9 months old (n = 26, n = 17), and 12 months old (n = 22, n = 16). (M) Contrast sensitivity threshold at different spatial frequencies (mean ± SEM) (number of animals [control versus experimental]): 3 months old (n = 11, n = 11), 6 months old (n = 10, n = 11), 9 months old (n = 11, n = 8), and 12 months old (n = 20, n = 11). *$p < 0.05$; **$p < 0.01$, ***$p < 0.001$. See also Figure S3.

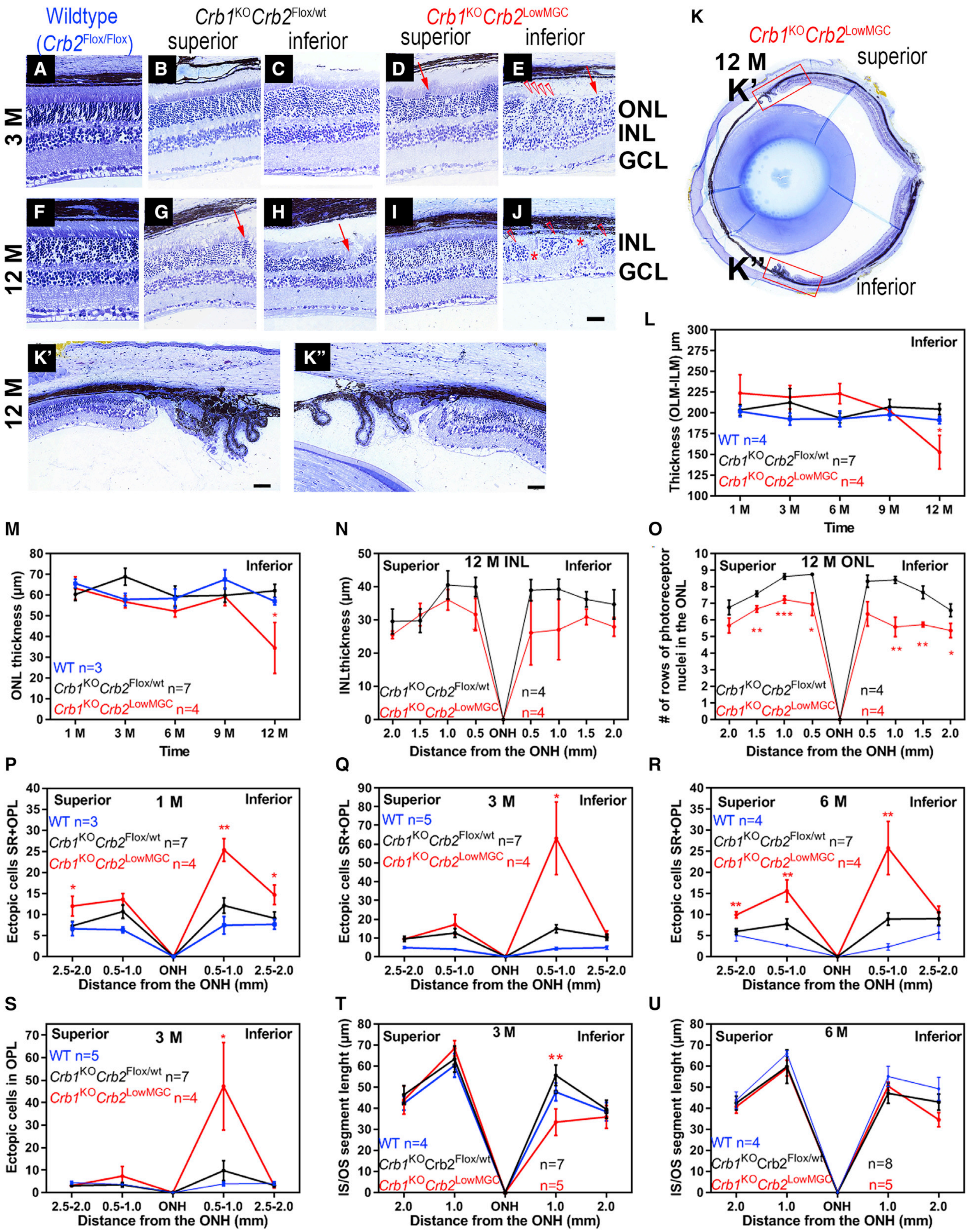

(legend on next page)

but it was not clear how much mCRB2 is contributed by MGCs and PRCs.[6] In this study, we semi-quantified mCRB2 protein expression (on fluorescence intensity) at the OLM and the number of OLM breaks in 3-month-old *Crb2*$^{\text{Flox/Flox}}$ (WT-like), *Crb2*$^{\Delta\text{Rods}}$ (ablation of *Crb2* in rods), *Crb1*^{KO}*Crb2*$^{\text{Flox/WT}}$, *Crb1*^{KO}*Crb2*^{LowMGC}, and *Crb1*^{KO}*Crb2*$^{\Delta\text{Rods}}$ mice. We validated our previous results, indicating that the OLM mCRB2 protein expression in the inferior retina compared to the superior retina was similar to the previous mixed genetic background of *Crb1*^{KO} mice.[28] The CRB2 protein expression was decreased at the OLM by 49% ± 3% (SEM) in *Crb2*$^{\Delta\text{Rods}}$ (n = 3 mice), 34% ± 10% in *Crb1*^{KO}*Crb2*^{LowMGC} (n = 5 mice), and 62% ± 12% in *Crb1*^{KO}*Crb2*$^{\Delta\text{Rods}}$ (n = 3 mice) compared to *Crb1*^{KO}*Crb2*$^{\text{Flox/WT}}$ retinas (n = 7), suggesting that MGCs contribute about half of the total CRB2 protein levels to the OLM (Figure 3Z). CRB2 protein expression between the peripheral (27 ± 6 AU [arbitrary fluorescence unit] and 15 ± 4 AU; n = 5 mice) and central retina (21 ± 1 AU and 14 ± 5 AU) was not statistically different in *Crb1*^{KO}*Crb2*$^{\text{Flox/WT}}$ (p = 0.08) or *Crb1*^{KO}*Crb2*^{LowMGC} mice (p = 0.30; data not shown).

The adherens junctions and the subapical region are located at the OLM. We and others have previously investigated the recruitment of adherens junction markers (e.g., cadherins or catenins) by the Crumbs complex at the subapical region.[8] The Crumbs complex consists of the CRB protein family (CRB1 and CRB2), the PALS1 (MPP5)-PATJ-MUPP1 protein complex, and the PAR6-PAR3-aPKC-CDC42 protein complex.[29–32] Disruption of the Crumbs complex leads to loss of polarity and loss of adhesion in many *Crb* mouse models.[5,7,8,12,16,28,33,34] Semi-quantification of p120-catenin, an adherens junction marker, showed a 28% ± 3% (SEM) decrease in *Crb2*$^{\Delta\text{Rods}}$ compared to WT and a 45% ± 7% reduction in *Crb1*^{KO}*Crb2*$^{\Delta\text{Rods}}$ compared to *Crb1*^{KO}*Crb2*$^{\text{Flox/WT}}$ mice (Figure 3AA). No statistical difference in p120-catenin expression was found between *Crb1*^{KO}*Crb2*$^{\text{Flox/WT}}$ and *Crb1*^{KO}*Crb2*^{LowMGC} mice (Figure 3AA).

Furthermore, the level of CRB1 and CRB2 proteins at the OLM determined the number of OLM breaks. More OLM breaks were found in mice with less CRB protein expression at the OLM (Figure 3BB). We further validated our previous results that the loss of CRB1 or CRB2 reduces the p120-catenin protein localization at the OLM, subsequently destabilizing the OLM and reducing adhesion between MGCs and photoreceptors, causing OLM breaks, and finally facilitating photoreceptor loss in the form of protrusion of photoreceptor nuclei through the OLM into the layer of the inner segments.[28] Finally, the morphological data at 3 months of age suggest that the *Crb1*^{KO}*Crb2*^{LowMGC} retinas show more degeneration in the superior as well as inferior retinal quadrants compared to *Crb1*^{KO}*Crb2*$^{\text{Flox/WT}}$ retinas. In the next section, we describe studies on the hypothesis of whether the MGCs in *Crb1*^{KO}*Crb2*^{LowMGC} retinas are more sensitive to the glial toxin DL-AAA than in littermate control *Crb1*^{KO}*Crb2*$^{\text{Flox/WT}}$ retinas.

Exposure to DL-AAA causes lasting retinal damage and worsened sight

A low dose of 100 μg of DL-AAA injected intravitreally in WT mice was shown by others to disrupt the OLM, causing photoreceptor nuclei protrusions into the photoreceptor segment layers that were resolved during 48 h.[17,35] In this study, we examined whether retinas with reduced levels of CRB2 proteins in *Crb1*^{KO}*Crb2*^{LowMGC} MGCs are more sensitive to DL-AAA than are retinas expressing normal levels of CRB2 proteins in *Crb1*^{KO}*Crb2*$^{\text{Flox/WT}}$ MGCs.

We challenged 2-month-old WT-like (*Crb2*$^{\text{Flox/Flox}}$) mice and the RP *Crb1*^{KO}*Crb2*$^{\text{Flox/WT}}$ and *Crb1*^{KO}*Crb2*^{LowMGC} mice with different doses of DL-AAA (100, 150, or 200 μg) and analyzed the retinal morphology, the retinal transmission (ERG), and vision-guided behavior (OKT) 1 month after intravitreal injection (3-month-old-mice; Figure 4A). No effect was seen on OKT and ERG when we injected PBS as a control (Figures 4B–4I). An overall dosage effect of DL-AAA on retinal transmission (ERG) and vision-guided behavior (OKT) was found for all mouse lines (Figures 4B–4I). 100 μg of DL-AAA markedly decreased the retinal transmission for the scotopic a-wave and b-wave (Figures 4C–4E) and the OKT thresholds (Figures 4F–4I) in the *Crb1*^{KO}*Crb2*^{LowMGC} mice but not in the *Crb2*$^{\text{Flox/Flox}}$ and *Crb1*^{KO}*Crb2*$^{\text{Flox/WT}}$ mice.

No effect was seen on morphology when we injected PBS as a control (Figures 4K–4N). Very little effect on morphology was found at 100 μg of DL-AAA except for the *Crb1*^{KO}*Crb2*^{LowMGC} retinas (Figures 4O–4S). The *Crb1*^{KO}*Crb2*^{LowMGC} retinas had an overall worsened retinal phenotype on the superior as well as the inferior retina as indicated by the ingressions (asterisks) and protrusions (arrow) in the retina (Figures 4R and 4S). 150 and 200 μg of DL-AAA caused irreversible retinal damage on morphology in all mouse lines (Figures 4T–4Z, 4AA, 4BB, and 4CC). The decrease in ERG/OKT and worse retinal morphology indicates that *Crb1*^{KO}*Crb2*^{LowMGC} retina might be more sensitive to OLM disruptions induced by DL-AAA. In the next section we investigated whether the increased sensitivity could be alleviated by h*CRB1* or h*CRB2* gene supplementation therapy.

Figure 2. Removal of CRB1 and low levels of CRB2 in MGCs lead to abnormal layering in the inferior quadrants

(A–K″) Toluidine-stained light microscopy of retinal sections from control (*Crb2*$^{\text{Flox/Flox}}$, *Crb1*^{KO}*Crb2*$^{\text{Flox/WT}}$, and *Crb1*^{KO}*Crb2*^{LowMGC}) mice at 3 and 12 months of age. Representative morphological changes: protrusions (red arrows downward), ingressions, neovascularization (asterisks), and loss of inner/outer segments of photoreceptors (red triangles). (K–K″) Representative 12-month-old retina of a *Crb1*^{KO}*Crb2*^{LowMGC} mouse indicates photoreceptor layer presence in periphery and superior quadrants of the retina. (L–U) Spidergrams of the retina of wild-type (WT; *Crb2*$^{\text{Flox/Flox}}$), *Crb1*^{KO}*Crb2*$^{\text{Flox/WT}}$, and *Crb1*^{KO}*Crb2*^{LowMGC} mice. (L and M) Decrease of retinal thickness (outer limiting membrane (OLM) to the inner limiting membrane) and outer nuclear layer (ONL) thickness at 1 mm distance on the inferior retina in 1-, 3-, 6-, 9-, and 12-month-old mice. (N and O) Inner nuclear layer (INL) thickness and the number of rows of photoreceptor nuclei in the ONL of 12-month-old mice. (P–S) Total number of ectopic cells at 1, 3, and 6 months of age. (S) Most ectopic cells are in the outer plexiform layer (OPL) at 3 months of age. (T and U) Inner/outer segment length of photoreceptors in the periphery (2.0 mm) and central (1.0 mm) from the optic nerve head (ONH). Scale bars, 50 μm. Data are presented as mean ± SEM. *p < 0.05, **p < 0.01, ***p < 0.001.

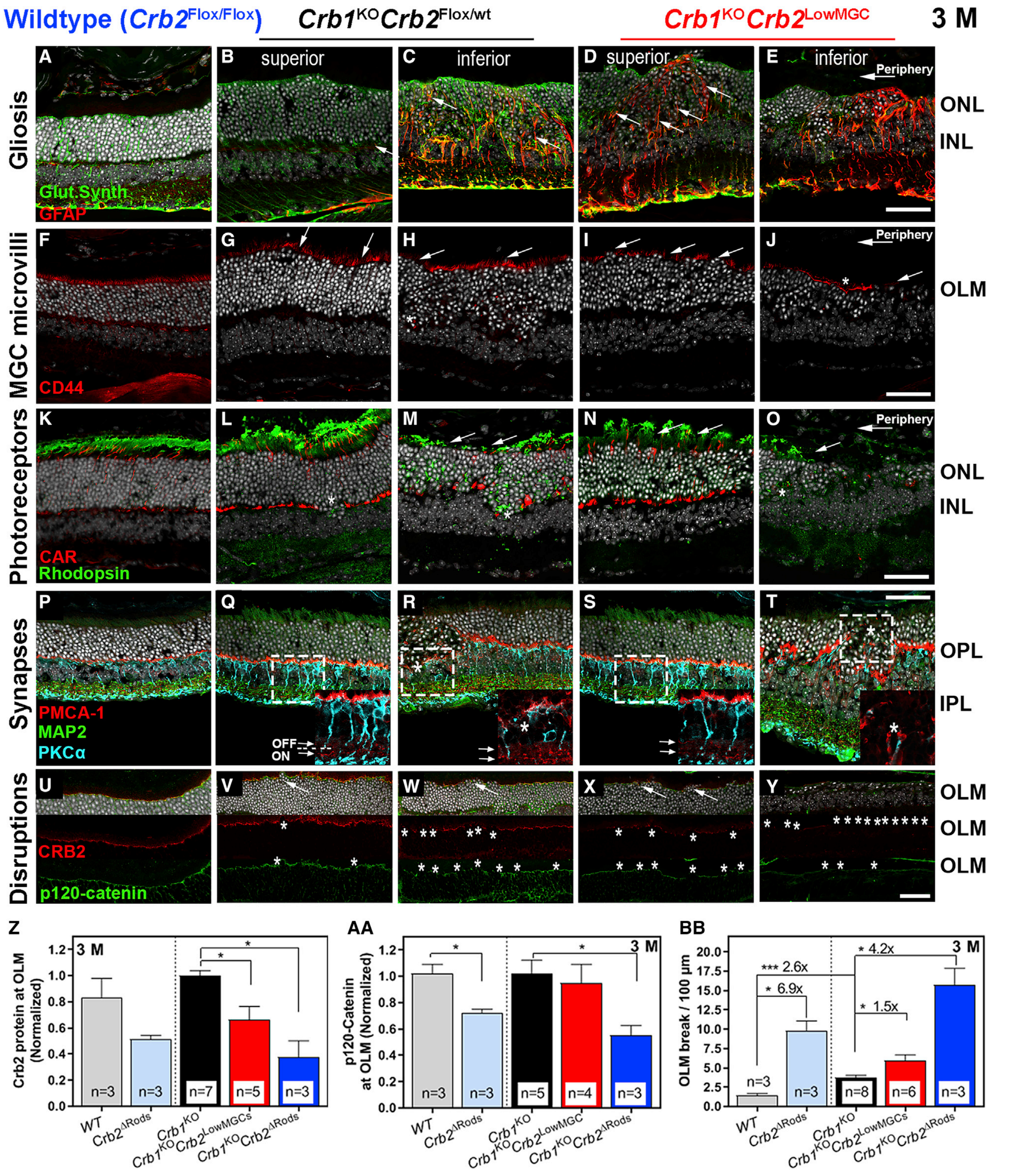

Figure 3. *Crb1*^{KO}*Crb2*^{LowMGC} retinas compared to *Crb1*^{KO}*Crb2*$^{Flox/WT}$ retinas show more disruptions at the OLM

Immunohistochemistry of 3-month-old mice. A representative image is shown of three to six retinas per group analyzed. (A–Y) Sections were stained for: (A–E) glutamine synthetase (green) for MGCs and glial fibrillary acidic protein (GFAP; red) for MGC stress fibers; (F–J) CD44 (red) for Müller glial microvilli processes; (K–O) cone arrestin (CAR; red) for cone photoreceptor segments and rhodopsin for rod photoreceptor outer segments (green); (P–T) microtubule-associated protein 2 (MAP2) (green) for ganglion cells

(legend continued on next page)

rAAV-h*CRB* protects retinal morphology, OKT, and ERG response in the DL-AAA-challenged *Crb1*^{KO}*Crb2*^{LowMGC} RP mouse model

We previously determined the tropism and potency of the ShH10^{Y445F} capsid (and the cell-specific expression of the cytomegalovirus [CMV] and minimal CMV [CMVmin] promoter) and the h*CRB1* codon-optimized or the h*CRB2* codon-optimized cDNA delivery to MGCs by intravitreal delivery of rAAVs in WT mice. We previously showed that the ShH10^{Y445F} capsid can effectively infect and efficiently express GFP in more than 40% of all mouse MGCs by intravitreal injection.[36,37] We also previously showed that subretinally injected rAAV serotype 2 (rAAV2)/9.CMVmin.h*CRB1*co.spA or intravitreal rAAV2/ShH10^{Y445F}.CMVmin.h*CRB1*co.spA can express hCRB1 protein at the OLM in *Crb1*^{KO} mice. Finally, we previously demonstrated that we can express hCRB2 protein at the OLM by intravitreal delivery of rAAV2/ShH10^{Y445F}.CMV.h*CRB2*co.spA in *Crb2* conditional KO (cKO) retinas.[13]

rAAV2/ShH10^{Y445F}.CMVmin.h*CRB1*co.spA (rAAV-h*CRB1*) or rAAV2/ShH10^{Y445F}.CMV.h*CRB2*co.spA (rAAV-h*CRB2*) was injected intravitreally into one eye of *Crb1*^{KO}*Crb2*^{LowMGC} mice at P21. Then, 2-month-old retinas were challenged by intravitreal injection of 100 μg of DL-AAA in both eyes. ERG, OKT, spectral domain optical coherence tomography (SD-OCT), and retinal morphology were assessed in 3-month-old mice (Figure 5A).

The overall retinal morphology improved with both rAAV vectors (Figures 5B–5G). Retinas receiving the rAAV-h*CRB* treatment had more rows of photoreceptor nuclei in the ONL (Figure 5H). The expression of hCRB in mouse MGCs protected against the protrusion of photoreceptor nuclei into the subretinal space and OPL (Figure 5I). In the central-inferior retina of *Crb1*^{KO}*Crb2*^{LowMGC} mice, the photoreceptor IS/OS length was 33 ± 6 μm (see also Figure 2P), 21 ± 6 μm in 100-μg DL-AAA-treated mice, 41.7 ± 5 μm in 100-μg DL-AAA+(rAAV-h*CRB1*)-treated mice, and 40 ± 22 μm in 100-μg AAA+(rAAV-h*CRB2*)-treated mice (Figure 5J), indicating a significant protection against loss of photoreceptor segment lengths in the central area for rAAV-h*CRB*-injected eyes. We then investigated the retinal morphology by SD-OCT (Figures 5K–5M). We found retinal degeneration in the central inferior quadrants at around 0.5–0.9 mm from the optic nerve head (ONH) on the volume intensity projection (VIP) in the ONL comparable to changes found on plastic sections (Figures 5H and 5J). More extensive disruptions in the OPL/ONL/OLM of control mice (100 μg of DL-AAA only) were found in the inferior and superior retinal quadrants (red arrows in Figures 5K–5M). Interestingly, some mice injected with rAAV-h*CRB1* showed many vitreous-infiltrating cells (asterisks in Figure 5L).

We measured ERG and OKT visual acuity and contrast sensitivity in these mice. We analyzed the differences between the rAAV-h*CRB*-treated eye against the eye not receiving the rAAV treatment (control eye) because the variation between mice was considerable, and comparison on an individual mouse reduces variation and permits a pairwise comparison, reducing the numbers of mice needed to show effects (see Figures 6 and S4 for absolute values). The retinal function measured by scotopic and photopic ERG was significantly higher in rAAV-h*CRB2*-treated eyes, but no significant changes were found in rAAV-h*CRB1*-treated eyes compared to control eyes (Figures 6A–6C; see also Figure S4). The visual acuity improved for rAAV-h*CRB2*-treated eyes but not for rAAV-h*CRB1*-treated eyes (Figure 6D). The contrast sensitivity threshold (spatial frequency: 0.032, 0.064, and 0.092) was significantly increased upon delivering h*CRB2* cDNA but not for h*CRB1* cDNA to MGCs (Figures 6E–6G; Figures S4N and S4O). The tracking at 0.092 cycles/degree was significantly worse for rAAV-h*CRB1*-injected eyes compared to control eyes (Figure 6G; Figures S4N and S4O).

rAAV-h*CRB* therapy reduces gliosis and protects Müller cell microvilli and photoreceptor IS/OS

MGCs extend from the endfeet at the ILM up to the apical villi above the OLM and support/maintain retinal homeostasis and retinal integrity. Long Müller glial stress fibers (GFAP-positive) extended through the retina in the inferior-central retinal areas of *Crb1*^{KO}*Crb2*^{LowMGC} mice injected with 100 μg of DL-AAA (Figure 7A, arrows indicate stress fibers). However, eyes treated by rAAV-h*CRB* showed shortened and less GFAP-positive stress fibers extending into the ONL (Figures 7B and 7C, arrows indicate stress fibers). Similarly, shortened and thickened Müller microvilli were observed in the retina of eyes injected with DL-AAA without rAAV-h*CRB* treatment. However, retinas with rAAV-h*CRB* treatment displayed less severely affected Müller microvilli in the inferior retinal quadrants (Figures 7E and 7F).

Crb1^{KO}*Crb2*^{LowMGC} retinas exposed to DL-AAA-mediated loss of adhesion had long stretches of no inner/outer photoreceptor segments in the inferior retinal quadrants stained by rhodopsin and cone arrestin (Figure 7G, arrows). Also, more ectopic nuclear rhodopsin expression in photoreceptor ingressions was observed (Figure 7G, asterisks indicate ingressions). The most affected inferior-central retinal quadrants frequently had some outer photoreceptor segments left in rAAV-h*CRB*-treated eyes compared to the control eyes (Figures 7G and 7I).

Retinas of *Crb1*^{KO}*Crb2*^{LowMGC} mice injected with DL-AAA had more ectopic synapses and loss of horseshoe-shaped synapses (Figure 7G).

and synapses in the inner plexiform layer (IPL),[26] PMCA1 (red) for pre-synapses of photoreceptors at the OPL and lamina a/b in the IPL,[27] and PKCα (light blue) for bipolar cells and bipolar post-synapses at the OPL; and (U–Y) subapical region marker CRB2 (red) and the adherens junction marker p120-catenin (green). (Z and BB) The decrease in CRB proteins and p120-catenin proteins at the OLM increases breaks at the OLM in 3-month-old WT, *Crb2*$^{\Delta Rods}$, *Crb1*^{KO}*Crb2*$^{Flox/WT}$, *Crb1*^{KO}*Crb2*^{LowMGC}, and *Crb1*^{KO}*Crb2*$^{\Delta Rods}$ mice. (Z) Normalized CRB2 protein expression to *Crb1*^{KO} mice (fluorescence). (AA) Normalized p120-catenin protein expression to *Crb1*^{KO}*Crb2*$^{Flox/WT}$. (BB) OLM breaks per 100 μm retinal length. GCL, ganglion cell layer. Scale bars, 50 μm; inserts, 50 μm. Data are presented as mean ± SEM. *p < 0.05, **p < 0.01, ***p < 0.001.

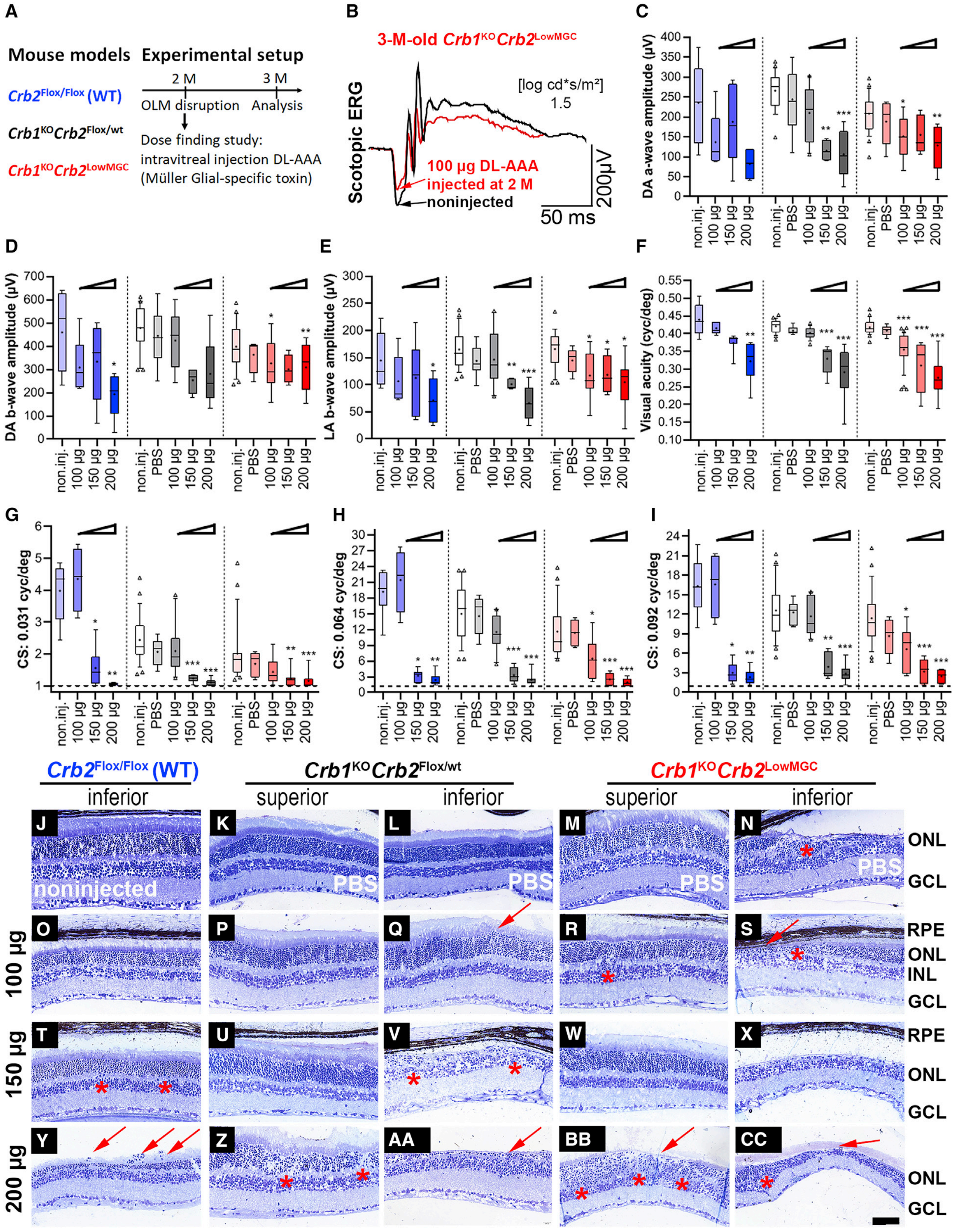

(legend on next page)

Retinas treated with rAAV-h*CRB* showed smaller disruptions of horseshoe-shaped synapses at the OPL (Figures 7G–7I; see also Figures 5K–5M, arrows). Also, DL-AAA-induced retinal stress may induce synaptic changes in the IPL but no differences between the previously described conditions without DL-AAA (Figures 3P–3T), with 100 μg of DL-AAA, or DL-AAA with rAAV-h*CRB* treatment were observed (Figures 7J–7L, asterisks indicate photoreceptor [synaptic] ingressions).

Neovascularization, activated microglial cells, ectopic h*CRB1* expression, and ciliary body changes related to rAAV-h*CRB1* intravitreal injections

rAAV-h*CRB1* treatment produced hCRB1 protein at the OLM (Figures S5A–S5E), but it was also found in the ciliary body (Figure S5D). Similarly, CRB2 was found at the OLM in rAAV-h*CRB2* of non-treated retinas (Figure S5G). The benefit of the rAAV-h*CRB2* over rAAV-h*CRB1* therapy on ERG retinal transmission and OKT behavior outcome measures (see Figure 6 and Figure S4) compared to similar benefits on retinal morphology (Figure 5) prompted us to further investigate whether one of these vectors increases neovascularization events or microglial activation.

First, we characterized the background of neovascularization and microglial activation in the inferior-central quadrants of 3-month-old $Crb2^{Flox/Flox}$, $Crb1^{KO}Crb2^{Flox/WT}$, and $Crb1^{KO}Crb2^{LowMGC}$ mice injected with DL-AAA (100 μg) against the noninjected control eyes (Figures S5H–S5M). A strong increase in neovascularization events was detected in the degenerate inferior retinal quadrants of $Crb1^{KO}Crb2^{LowMGC}$ mice injected with DL-AAA (100 μg) upon immunohistochemical staining for plasmalemma vesicle-associated protein (PLVAP; a marker for early vascular leakage[40,41]) or activated microglial cells (CD11b-positive microglial cell dendrites and migration to the ONL and GCL) (Figure S5M). Neovascularization was not observed in the RPE, ONL, or INL when rAAV-h*CRB1* or rAAV-h*CRB2* was administered to $Crb1^{KO}Crb2^{LowMGC}$ mice (Figures S5N and S5O), matching the proper retinal lamination seen in four eyes on plastic sections and five to six eyes per rAAV-vector on SD-OCT morphology (Figure 5). However, we also observed many PLVAP-positive ectopic cells in the lower part of the ganglion cell layer (GCL)/nerve fiber layer (NFL) intermingled with activated microglial cells (CD11b-positive) in three out of six eyes on immunohistochemistry when rAAV-h*CRB1* was injected in $Crb1^{KO}Crb2^{LowMGC}$ mice (Figure S5N, arrows indicate PLVAP-positive cells, asterisk indicates microglial activation in the GCL; see also Figure 5L SD-OCT image, arrows indicate massive cell infiltration in the vitreous body). Neovascularization events were only seen in one eye out of four eyes and no microglial activation in four eyes sampled on immunohistochemistry of $Crb1^{KO}Crb2^{LowMGC}$ mice injected with rAAV-h*CRB2* (data not shown). No thickened GCL and NFL with PLVAP-positive cell nuclei were found in control eyes injected with DL-AAA (100 μg) in $Crb1^{KO}Crb2^{LowMGC}$ mice (Figure S5M), indicating that rAAV-h*CRB1* may increase neovascularization events in the GCL/NFL. We also found double-positive-labeled PLVAP and vascular endothelial cadherin (VE-cadherin)-expressing vascular cells in the ciliary body of $Crb1^{KO}Crb2^{LowMGC}$ mice treated with rAAV-h*CRB1* but not in the rAAV-h*CRB2* treatment group (Figures S5P–S5S). rAAV-vector contaminations could explain the neovascularization events seen in the rAAV-h*CRB1* treatment group. However, we did not detect major protein contaminants in two independently produced rAAV batches used in the study (Figure S5T).

In summary, we observed more neovascularization events with immunohistochemistry in the ONL/OPL/INL when 100 μg of DL-AAA was injected intravitreally in $Crb1^{KO}Crb2^{LowMGC}$ mice compared to noninjected $Crb1^{KO}Crb2^{LowMGC}$ mice (Figures S5L and S5M). Fewer neovascularization events in the ONL/OPL/INL were observed with immunohistochemistry and SD-OCT in the rAAV-h*CRB2* treatment group or rAAV-h*CRB1* treatment group compared to the control eyes. However, we observed in the treatment groups of rAAV-h*CRB2* compared to rAAV-h*CRB1* fewer GFAP-positive stress fibers (Figures 6B and 6C) and fewer neovascularization events in the GCL/NFL (Figures S5H–S5O). Additionally, the rAAV-h*CRB1* treatment group showed consistent neovascularization at the ciliary body that was not found in control eyes (100 μg of DL-AAA injected) or in rAAV-h*CRB2*-treated eyes. Neovascularization at the ciliary body was previously observed after 4.5 months after intravitreal injection of rAAV-h*CRB1* but not by rAAV-h*CRB2* in $Crb1^{KO}Crb2^{Flox/WT}$Chx10$CreGFP^{Tg/+}$ retinas.[13]

DISCUSSION

In this study, we show that (1) in $Crb1^{KO}Crb2^{LowMGC}$ mice, a minimum of half of the endogenous mCRB2 levels in MGCs lacking

Figure 4. Intravitreal injection of DL-AAA worsens retinal morphology, retinal transmission, and vision-guided behavior

(A) Mouse models ($Crb2^{Flox/Flox}$, $Crb1^{KO}Crb2^{Flox/WT}$, $Crb1^{KO}Crb2^{LowMGC}$) were exposed to OLM disruptions by DL-AAA intravitreal injection of DL-AAA at 2 months, and the effect was measured at 3 months (ERG, OKT, morphology). (B) Single-flash scotopic ERG traces at 1.5 log cd·s/m^2 intensity for a $Crb1^{KO}Crb2^{LowMGC}$ mouse (black trace indicates noninjected; red trace indicates 100 μg of DL-AAA injected). (C–E) $Crb2^{Flox/Flox}$, blue boxplots; $Crb1^{KO}Crb2^{Flox/WT}$, gray boxplots; $Crb1^{KO}Crb2^{LowMGC}$, red boxplots. (C) Scotopic a-wave (μV) at 1.5 log cd·s/m^2. (D) Scotopic b-wave (μV) and photopic b-wave (μV) at 1.5 log cd·s/m^2. Number of animals for ERG (C–E): $Crb2^{Flox/Flox}$: noninjected, n = 8; 100 μg, n = 5; 150 μg, 200 μg, n = 5 per group. $Crb1^{KO}Crb2^{Flox/WT}$: noninjected, n = 23; PBS, n = 7; 100 μg, n = 10; 150 μg, n = 4; 200 μg, n = 6. $Crb1^{KO}Crb2^{LowMGC}$: noninjected, n = 24; PBS, n = 5; 100 μg, n = 9; 150 μg, n = 5; 200 μg, n = 7. (F–I) Optokinetic head-tracking responses (OKT). (F) Spatial frequency threshold (visual acuity). (G–I) Contrast sensitivity threshold at 0.031, 0.064, and 0.092 spatial frequency (cycles/degree). Number of animals for OKT (F–I): $Crb2^{Flox/Flox}$: noninjected, n = 6; 100 μg, n = 4; 150 μg, n = 8; 200 μg, n = 8. $Crb1^{KO}Crb2^{Flox/WT}$: noninjected, n = 25; PBS, n = 7; 100 μg, n = 10; 150 μg, n = 6; 200 μg, n = 7. $Crb1^{KO}Crb2^{LowMGC}$: noninjected, n = 25; PBS, n = 4; 100 μg, n = 9; 150 μg, n = 7; 200 μg, n = 8. (J–Z, AA, BB, and CC) Toluidine-stained light microscopy of retinal sections from 3-month-old mice. Scale bars, 50 μm. Data are presented as boxplots (10%–90%) and outliers (triangles). Mean is indicated as a plus sign (+). An ANOVA (Kruskal-Wallis), followed by a Bonferroni post hoc test, was performed to determine statistical significance comparing the noninjected (non.inj.) values to PBS and 100 μg, 150 μg, and 200 μg of DL-AAA-injected mouse values. *$p < 0.05$, **$p < 0.01$, ***$p < 0.001$.

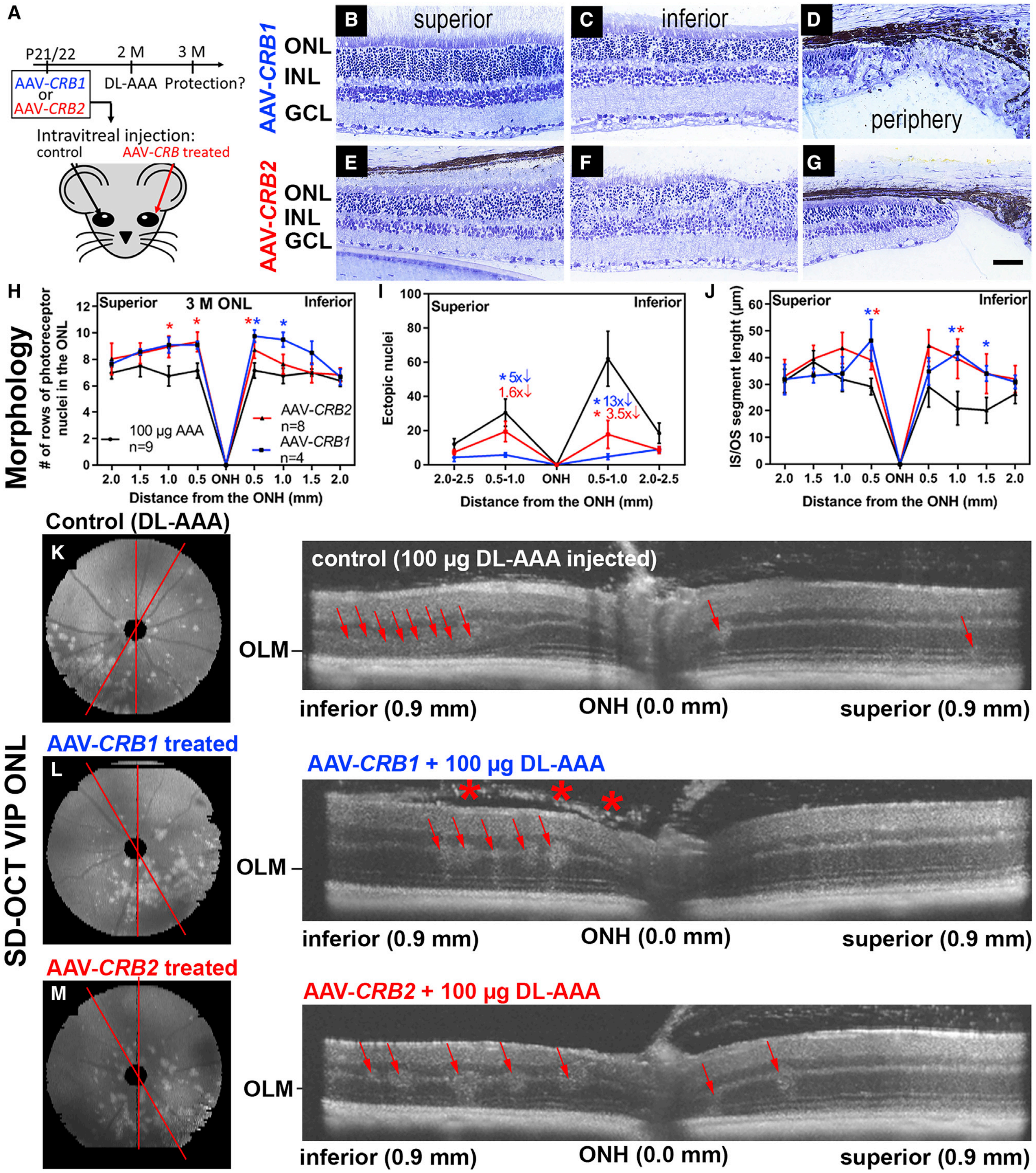

Figure 5. rAAV-h*CRB2* protects against retinal disorganization and degeneration in the AAA-challenged *Crb1*^{KO}*Crb2*^{LowMGC} mice

(A) Retinitis pigmentosa model: rAAV-h*CRB* was injected in one eye versus noninjected eye (control) at P21/P22. Both eyes were then injected with a MGC-specific OLM stressor (100 µg of DL-AAA) at the 2-month time point. The potential rAAV therapy protective effect was measured at 3 months. (B–G) Toluidine-stained light microscopy of retinal sections from 3-month-old *Crb1*^{KO}*Crb2*^{LowMGC} mice that were treated with (B–D) rAAV2/ShH10^{Y445F}.CMVmin.h*CRB1* or treated with (E–G) rAAV2/ShH10^{Y445F}.CMV.h*CRB2*. (H–J) Spidergrams of the retina of *Crb1*^{KO}*Crb2*^{LowMGC} mice (100 µg of AAA injected, n = 9; rAAV-h*CRB1* injected, n = 4; and rAAV-h*CRB2* injected, n = 8). (H–J) rAAV-h*CRB* treated retinas had more rows of photoreceptor nuclei in the ONL (H), fewer ectopic photoreceptor cell nuclei in the subretinal space and

(legend continued on next page)

CRB1 (with normal levels of CRB2 expressed at the OLM in photoreceptors) results in a RP retinal phenotype. Interestingly, in previous studies we showed that complete loss of CRB2 in MGCs lacking CRB1 resulted in an LCA retinal phenotype.[5] (2) Mice with reduced levels of CRB2 protein in MGCs lacking CRB1 in MGCs (*Crb1*^{KO} *Crb2*^{LowMGC}) showed increased sensitivity to OLM disruptions upon exposure to DL-AAA. (3) rAAV-h*CRB2* therapy to MGCs protects against OLM disruptions, a decrease of ERG responses, and a loss of OKT contrast sensitivity in *Crb1*^{KO}*Crb2*^{LowMGC} mice.

CRB1-related RP patients have a nonfunctional or less functional CRB1 protein in MGCs and photoreceptors.[4] We have shown that we can model the *CRB* phenotype in human retinal organoids *in vitro*[4] and in mice *in vivo*. In the present study, we further explored the effect of ablating *CRB* in late-born retinal cells, such as MGCs. Previously, we showed that the full ablation of *Crb1* and *Crb2* in MGCs (*Crb1*^{KO}*Crb2*$^{\Delta MGC}$) caused a severe LCA-like retinal phenotype with no ERG response already in 1-month-old mice.[5] Previously, we showed that full ablation of *Crb2* specifically in MGCs (*Crb2*$^{\Delta MGC}$) causes a slow progressing RP-like phenotype with sporadic disruptions at the OLM with protrusion of photoreceptor nuclei into the photoreceptor segment layer, similar to previous observations in retinas lacking CRB1, without effects on the ERG response.[5] In this study, we show that *Crb1*^{KO}*Crb2*^{LowMGC} mice develop a more severe RP-like phenotype than do *Crb1*^{KO} or *Crb2*$^{\Delta MGC}$ mice with clear effects on ERG and OKT responses and retinal morphology, suggesting similar functions of CRB1 and CRB2 proteins in MGCs.

Mice are housed under standard low light conditions that do not resemble the retinal stress that RP patients undergo in regular life. We have shown previously that light exposure can worsen the *CRB1* RP mouse phenotype.[8,12] Bright light exposure causes prolonged inflammation, neovascularization, and retinal damage in RP mouse models. Blue light exposure induces retinal degeneration, oxidative stress, and neuroinflammatory activity similar to dry age-related macular degeneration (AMD). In this study, we explored intravitreal DL-AAA injections that act on glial cells such as MGCs, causing disruptions at foci at the OLM. Since exposure of DL-AAA to the mouse retina, or loss of CRB1 or loss of CRB2 proteins in the mouse retina, causes disruptions at foci at the OLM, we examined whether mice with decreased levels of CRB proteins in MGCs are more sensitive to DL-AAA exposure than retinas with higher levels of CRB proteins in MGCs. The OLM disruptions caused by low doses of DL-AAA are reversible in WT mice.[17] The phenotype described indicated some similarities such as photoreceptor nuclei protrusions into the photoreceptor segment layer that we previously observed in *Crb1* RP mouse models. However, it was not known how DL-AAA could affect the visual tracking thresholds (OKT), the retinal function (ERG), quantitative retinal morphology, or mouse models with reduced cell adhesion. West et al.[17] showed that low levels of DL-AAA (100 μg) did not affect the gross retinal morphology in WT mouse retinas. In this study, we first demonstrated that mice expressing normal levels of endogenous CRB2 proteins in MGCs (*Crb2*$^{Flox/Flox}$ or *Crb1*^{KO}*Crb2*$^{Flox/WT}$) are less sensitive to exposure of 100 μg of DL-AAA than are mice with reduced levels of endogenous CRB proteins in MGCs (*Crb1*^{KO}*Crb2*^{LowMGC}). Subsequently, we demonstrated that increasing the levels of recombinant hCRB2 in MGCs in *Crb1*^{KO}*Crb2*^{LowMGC} retinas prior to exposure to DL-AAA decreased the sensitivity to DL-AAA exposure. We applied the rAAV-h*CRB* gene therapy vectors at P21, and it could have been more effective to apply rAAV-h*CRB* at earlier time points. In this study, we show that rAAV-h*CRB2* applied at P21 to *Crb1*^{KO}*Crb2*^{LowMGC} MGCs at early stage retinal disease effectively prevents against the adverse effects of exposure of DL-AAA. The *Crb1*^{KO} *Crb2*^{LowMGC} mice pre-treated intravitreally with rAAV-h*CRB2* and subsequently exposed to DL-AAA showed significantly fewer disruptions at the OLM, fewer protrusions of photoreceptor nuclei in the photoreceptor segment layers, less loss of photoreceptors, an improved contrast sensitivity as measured by OKT, and an improved retinal function as measured by ERG.

Intravitreal injection of rAAV vectors is more efficient in supplementing cDNA to MGCs compared to subretinal injections. For example, we have shown that rAAV2/ShH10^{Y445F} can effectively infect and efficiently express GFP in more than 40% of all mouse MGCs.[36,37] Yet, intravitreal injections increase the risk of alternate rAAV vector biodistribution and ectopic vector expression.[13] Others have indicated a transient inflammation of the aqueous and the vitreous body by empty rAAV capsids and rAAV supplementation vectors at high doses.[42] In this study, we re-assessed the effect of rAAV-h*CRB1* and rAAV-h*CRB2* to the vitreous body, ciliary body, and the neuroretina. We previously found that rAAV-h*CRB1* applied at P14 causes ectopic CRB1 expression within the epithelium of the ciliary body and the iris epithelia, affecting the corneal thickness, the eyeball perimeter, and CD11b- and CD3-positive infiltrating cells to the ciliary body.[13] We found similar ectopic rAAV-h*CRB1* expression at 3 months of age in the *Crb1*^{KO}*Crb2*^{LowMGC} mouse model injected at P21 with rAAV-h*CRB1* and at 2 months of age with DL-AAA injected. Interestingly, cDNA supplementation of h*CRB1* to MGCs (by rAAV capsids) improved the retinal morphology, such as a decrease in ectopic cells in the OPL/subretinal space and the maintenance of the number of rows of photoreceptor nuclei in ONL in the central retina.

We hypothesize that hCRB1 applied to Müller cells can protect against loss of OLM integrity, but OKT visual behavioral and ERG

OPL (I), and longer inner/outer segments of photoreceptors (J). (K–M) Representative spectral domain optical coherence tomography (SD-OCT) images indicate the phenotype in inferior-temporal quadrant in the volume intensity projection (VIP) of the ONL and representative radial SD-OCT averaged stacks (150°–180° or 180°–210°, see red lines) of superior-inferior retina indicate more retinal damage in eyes not supplemented with h*CRB1* or h*CRB2* cDNA (number of animals: rAAV-h*CRB1*, n = 5; rAAV-h*CRB2*, n = 6; untreated, n = 11). Arrows indicate ingressions; asterisks indicate infiltrating vitreous cells. IS/OS, inner/outer segments of photoreceptors. Scale bars, 50 μm. Data are presented as mean ± SEM. *p < 0.05, **p < 0.01, ***p < 0.001.

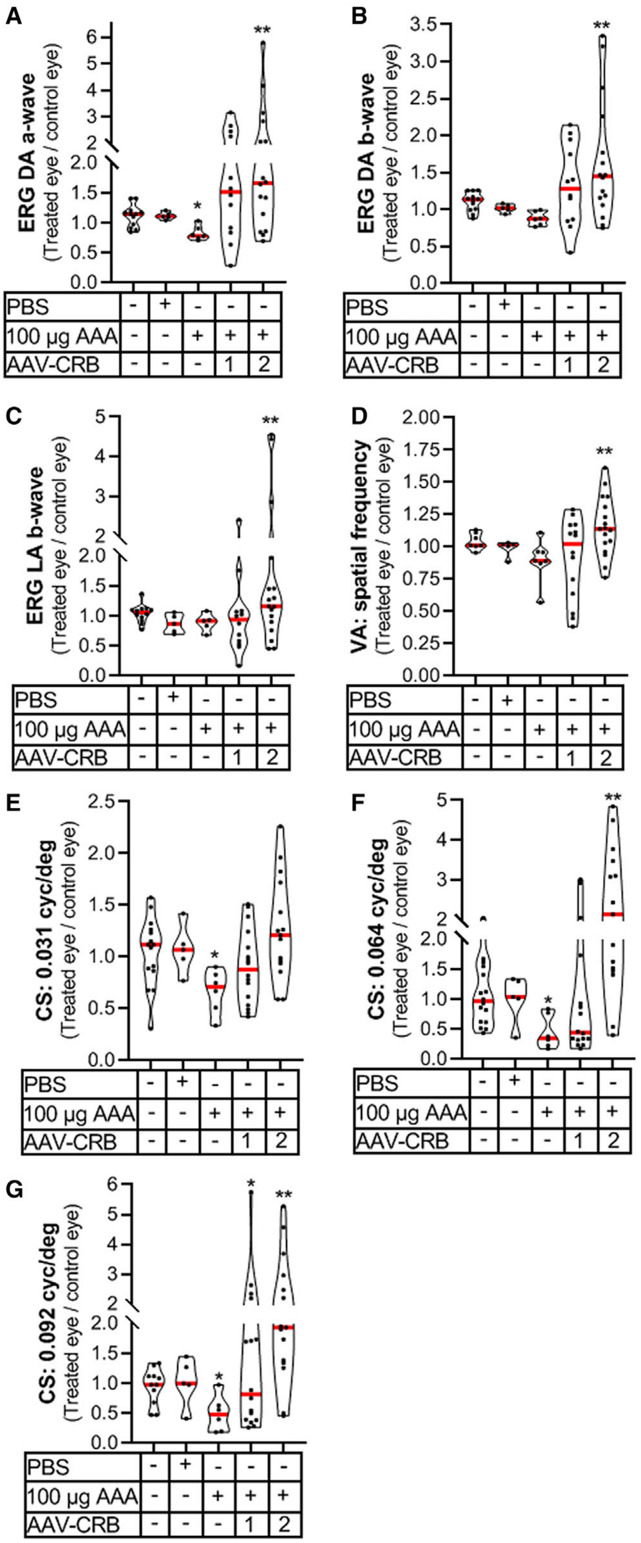

Figure 6. rAAV-h*CRB2* protects against loss of ERG and OKT response in AAA-challenged *Crb1*^{KO}*Crb2*^{LowMGC} mice

Group comparisons (e.g., rAAV-treated eye divided by control eye value) in violin plots in columns 1–5: (1) overall control: left eye value divided by the right eye value; (2) one eye injected with PBS divided by one eye not injected; (3) one eye injected with 100 μg of DL-AAA divided by one eye not injected; (4 and 5) P21/P22 injection of rAAV-h*CRB* in one eye, 100 μg of DL-AAA injected at 2 months in both eyes, and protection against retinal damage analyzed at 3 months (rAAV-treated divided by control eye). (A–C) Single-flash ERG traces at 1.5 log cd·s/m^2 intensity for the (A) scotopic a-wave, (B) scotopic b-wave, and (C) photopic b-wave. (D–G) Optokinetic head-tracking responses (OKT). (D) Spatial frequency threshold (visual acuity [VA]). (E–G) Contrast sensitivity threshold at 0.031, 0.064, and 0.092 spatial frequency (cycles/degree). The probability distribution is presented in a violin plot. The median is given in red. Dots in graphs represent the values obtained for each mouse. For statistical comparison, a paired t test on the total value of the rAAV-treated eye against total value of the eye not receiving the *CRB1* or *CRB2* cDNA (control eye) was performed. Mice tested are represented as black dots in figures. *p < 0.05, **p < 0.01, ***p < 0.001. See also Figure S4.

electrical transmission studies suggest that the neural network is not sufficiently restored, whereas morphological studies of the treated eyes suggest that there are adverse effects upon ectopic expression of CRB1. Potentially, the neovascularization events seen in the GCL/NFL of the inferior mouse retinas and the poor ERG/OKT responses measured in the rAAV-h*CRB1*-treated eyes, but not in the rAAV-h*CRB2* treated eyes, may be linked to differences of h*CRB2* over h*CRB1* (over-)expression in (1) MGCs, (2) astrocytes, (3) protein-protein interactions, or (4) immunogenic properties of hCRB1 protein in a *Crb1*^{KO} mouse. We discuss these points below.

(1) MGCs contribute to the maintenance and rigidity of the retinal layers (i.e., tensile strength), regulate blood flow and maintain the retina-blood barrier (e.g., release vascular endothelial growth factor [VEGF]), ensheathe synapses in IPL and the OPL, guide light rays to the segment layer, and remove waste products.[43–45] Mouse *Crb1* (m*Crb1*) and *Crb2* (m*Crb2*) expression levels in MGCs are linked to the intermediate capillary plexus development by suppressing the angiogenic growth factor *VEGFA*[46] and promoting *MMP-3* expression (extracellular matrix remodeling).[47] h*CRB1* expression protected MGCs less than did h*CRB2* from gliosis. (2) Astrocytes wrap around retinal endothelial cells and pericytes of the superficial capillary plexus at the GCL/NLF. The astrocytic foot processes may release angiogenic growth factors (e.g., VEGF) during retinal development and disease, altering the expression of tight junction proteins in retinal endothelial cells (blood-retinal barrier, ZO-1/occludin/VE-cadherin proteins).[48–50] VEGF suppression speeds up programmed capillary regression during development.[51] The AAV6 capsid variant ShH10^{Y445F} used in our study efficiently infects astrocytes.[52] We found only neovascularization events in the GCL/NFL with rAAV-h*CRB1* that may be linked to differences of h*CRB1* over h*CRB2* expression in DL-AAA-stressed astrocytes. (3) The protective function of hCRB2 over hCRB1 protein may also be explained by unknown differences in the intracellular protein function (both have short intracellular FERM/PDZ/ERLI domains[9,14]) or differences in hCRB homodimerization/heteromerization of extracellular hCRB/mCRB protein at the OLM. However, both vectors

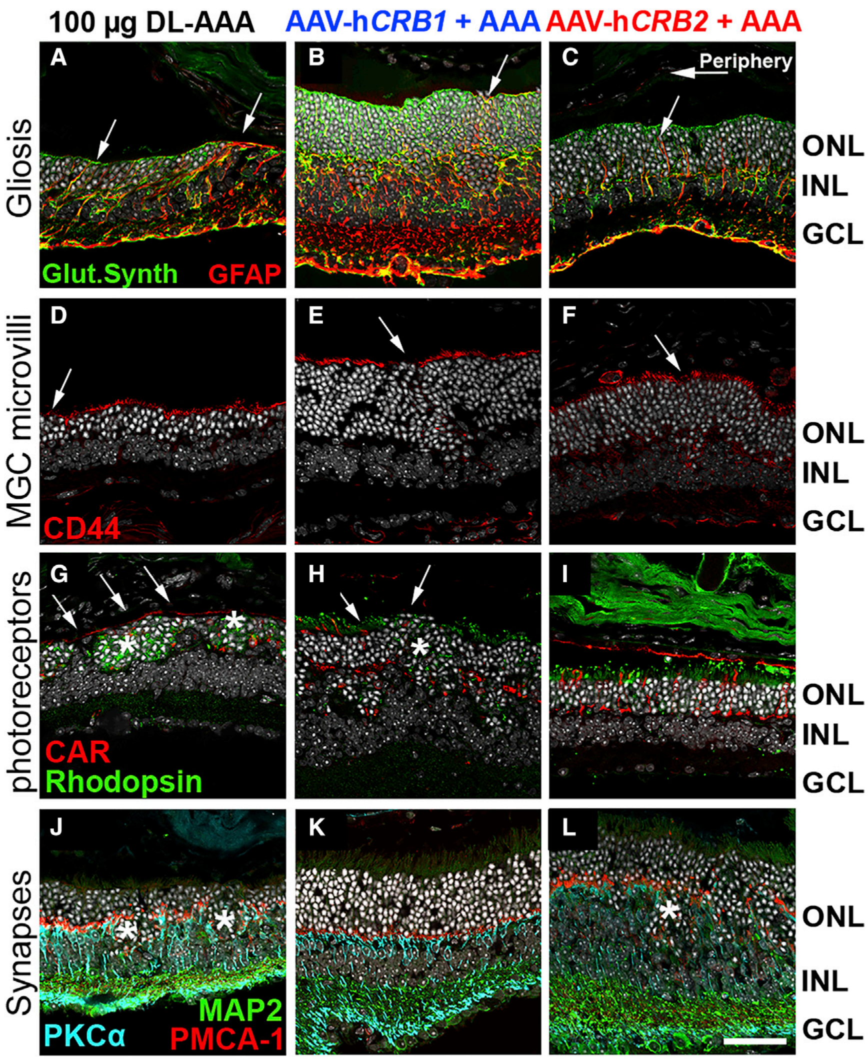

Figure 7. Intravitreal injection of rAAV-h*CRB* in the AAA-challenged *Crb1*^{KO}*Crb2*^{LowMGC} mouse model protects against loss of Müller glial microvilli length

Immunohistochemistry on the inferior retinal quadrants of 3-month-old mice that received an intravitreal injection of rAAV-h*CRB* in one of the two eyes at P21, and subsequently at 2 months of age intravitreal injections of 100 μg of DL-AAA in both eyes. (A–I) Sections were stained for: (A–C) glutamine synthetase (green) for Müller glial processes and GFAP (red) for stress fibers (arrows); (D–F) CD44 (red) for Müller glial microvilli processes subapical region marker (arrow indicates loss/decrease of villi); (G–I) CAR (red) for cone photoreceptor segments and rhodopsin for rod photoreceptors (green; arrows indicate loss/decrease in length of inner/outer photoreceptor segments; asterisks indicate intracellular rhodopsin expression in stressed rod photoreceptors); and (J–L) MAP2 (green) for ganglion cells, synapses in the IPL, as well as PRC inner segments;[26,38] PMCA1 (red) for synaptic elements of photoreceptors at the OPL and lamina a/b in the IPL;[27,39] and PKCα (light blue) for pre-synaptic elements of bipolar cells (asterisks indicate decrease in horseshoe-shaped synapse at OPL). Scale bars, 50 μm; inserts, 50 μm. 3–4 eyes per group were analyzed.

rescued the retinal morphology at the OLM where we would expect that the homodimerization/heteromerization takes place. (4) The *Crb1*^{KO}*Crb2*^{LowMGC} mice are naive for full-length mCRB1 protein but not for mCRB2 (expressed in, e.g., RPE cells, the retina, choroid, lung tissue, and the brain[5,53–55]), and the expression of hCRB1 on a *Crb1*^{KO} background may induce an immune reaction, whereas due to endogenous mCRB2 expression the retinal expression of hCRB2 is less likely to induce an immune reaction, especially in a *CRB1*-RP model with a leaky blood-retina barrier (partially aggravated by DL-AAA), allowing more infiltration of immune cells. More research is needed to delineate the effects of hCRB1 overexpression in MGCs or astrocytes on retinal vasculature in the healthy, degenerate, and *Crb1*^{KO} retina.

Nevertheless, the additive effect of DL-AAA on *Crb1*^{KO}*Crb2*^{LowMGC} MGCs may model the exudative vasculopathy seen in RP-*CRB1* patients[1] and help to better understand what therapies on MGC-regulated vasculopathy may be beneficial for RP-*CRB1* patients. Coats-like exudative vasculopathy (also called idiopathic retinal telangiectasia) is strongly associated with the RP-*CRB1* phenotype seen in clinics.[1] We observed (early) vascular leakage (PLVAP) in the inner retina in this study, but also previous studies found neovascularization (VEGF, von Willebrand factor [vWF], Fluorescein angiography-cSLO) in RP-*CRB1* mouse and rat models.[5,8,12,15,28,47,56] Subretinal injection of a high dose of DL-AAA induces MGC injuries that can develop into vascular telangiectasia and hemorrhages of the inner retinal vasculature in rats, rabbits, and nonhuman primates.[20,57,58] Thus, a (pan-) *CRB1* therapy may need to address not only the cell-cell adhesion at the OLM but also regulation of glial cells on retinal vasculature signaling and overall retinal maintenance.

We show that rAAV-h*CRB2* does not cause adverse changes at the ciliary body or the GCL/NFL. Furthermore, rAAV-h*CRB2* protected against loss of retinal and visual function and retinal morphology. Our study suggests that expression of hCRB2 does not cause adverse effects in mouse retinas and that it can significantly increase retinal adhesion in a new cell-adhesion *CRB1* RP mouse model. The study further strengthens the hypothesis that rAAV-h*CRB2* retinal gene therapy might be of benefit for RP patients with mutations in the *CRB1* gene.

MATERIALS AND METHODS

Mice

Procedures concerning animals were performed according to the Dutch Central Commission Animal Experimentation (CCD) license

no. AVD1160020172924, the working protocols (OZP nos. PE.18.016.002, PE.18.016.006, PE.18.016.007, and PE.18.016.010) approved by the Local Ethics Committee (Instantie voor Dierenwelzijn [IvD]) of the Leiden University Medical Center, and the Association for Research in Vision and Ophthalmology (ARVO) statement for the use of animals in ophthalmic and vision research. All mice used were maintained on a 99.9% C57BL/6JOlaHsd genetic background with a 12-h light/12-h dark cycle (standard low light housing condition ~10–20 lux) and supplied with food and water *ad libitum*. All experiments were carried out in male and female mice. All mouse strains below were confirmed to be *Crb1*^{rd8}-negative, *Nnt(exon 7–11)*$^{WT/WT}$, *Mmrn1(exon 8)*$^{-/-}$, α-synuclein(exon 6)$^{-/-}$, and *Pde6b*$^{WT/WT}$, thus similar to the genetic background of C57BL/6JOlaHsd mice.

Crb1^{KO}*Crb2*$^{Flox/Flox}$ mice were crossed with a *Crb1*^{KO}Pdgfrα-*Cre*$^{Tg/+}$ to produce *Crb1*$^{-/-}$*Crb2*$^{Flox/WT}$Pdgfrα-*Cre*$^{Tg/+}$ mice (*Crb1*^{KO} *Crb2*^{LowMGC}) and *Crb1*$^{-/-}$*Crb2*$^{Flox/WT}$ mice.[5,28] The *Crb1*^{KO} *Crb2*^{LowMGC} mice lack mCRB1 in radial glial progenitor cells and MGCs and reduced mCRB2 protein expression in MGCs during early retinal development.[5] The *Crb1*$^{-/-}$*Crb2*$^{Flox/WT}$Pdgfrα-*Cre*$^{Tg/+}$ mice contain a Pdgfrα-*Cre* transgene (C57BL/6-Tg(Pdgfrα-*cre*)1Clc/J) driving the *Cre* gene specifically in MGCs.[5,59] We determined the *Cre* mosaicism in a reporter mouse line (ROSA$^{mT/mG}$). We found that 95% of all MGCs excised membrane-targeted enhanced green fluorescent protein (mG) by Pdgfrα-Cre-mediated recombination.[5] mCrb2 protein is expressed by MGCs and PRCs at the OLM. The *Cre* mice used express the Cre recombinase specifically in MGCs, generating hemizygote *Crb2* MGCs. Assuming that 49% ± 3% (SEM) of mCRB2 is localized at the OLM within photoreceptors (Figure 3Z), we estimate that 51% of mCRB2 is localized at the OLM in control *Crb1*^{KO}*Crb2*$^{Flox/WT}$ MGCs. Since the total levels of mCRB2 at the OLM of *Crb1*^{KO}*Crb2*^{LowMGC} retinas are reduced by 34% ± 10% (SEM) compared to the reference littermate control *Crb1*^{KO}*Crb2*$^{Flox/WT}$ (*Crb1*^{KO}) retinas (Figure 3Z), we estimate that the levels at the OLM of mCRB2 in *Crb1*^{KO}*Crb2*$^{Flox/WT}$ MGCs dropped from 100% to 33% ([(51% − 34%)/51%] × 100 = 33%) specifically in *Crb1*^{KO}*Crb2*^{LowMGC} MGCs. The *Crb2*$^{\Delta Rods}$ and *Crb1*^{KO}*Crb2*-$^{\Delta}$Rods were previously described.[11] The mice contained a Rho-i*Cre* transgene ablating the *Crb2* gene in developing rod photoreceptors. Chromosomal DNA isolation and genotyping were performed as previously described.[5] All mice were euthanized using CO_2/O_2. The experimental and control mice were collected at the same time at 5–8 h within the light cycle to have comparable lengths of the IS/OS of photoreceptors.

ERG

Dark and light-adapted ERGs were performed under dim red light using an Espion E^2 (Diagnosys, Lowell, MA, USA). ERGs were performed on 1-, 3-, 6-, 9-, and 12-month-old *Crb1*^{KO}*Crb2*^{LowMGC} and *Crb1*^{KO} mice. The ERG values of the right and left eye were averaged for the analysis in Figures 1 and S2. One eye was used for analysis for other experiments (treatment versus control eye). Mice were anesthetized using 100 mg/kg ketamine and 10 mg/kg xylazine intraperitoneally, and the pupils were dilated using atropine drops (5 mg/mL). ERGs were recorded as previously described.[16] Scotopic recordings were obtained at −4, −3, −2, −1, 0, 1, 1.5, and 1.9 log cd·s/m^2 light intensity. Flicker recordings were obtained at 0.5 log·cd s/m^2 fixed light intensity at the frequencies 0.5, 1, 2, 3, 5, 7, 10, 12, 15, 18, 20, and 30 Hz. Photopic recordings were obtained at 30 cd/m^2 background light at −2, −1, 0, 1, 1.5, and 1.9 log·cd s/m^2 light intensity. The ERG tests were performed consecutively as follows: (1) scotopic, (2) flicker, (3) 10-min light exposure (30 cd·s/m^2 light intensity), and (4) photopic.

SD-OCT imaging

Mice were anesthetized using 100 mg/kg ketamine and 10 mg/kg xylazine intraperitoneally, and the pupils were dilated using atropine drops (5 mg/mL). The mouse was placed on a rodent alignment system with a bit bar (AIM-RAS, Bioptigen, USA). The optic nerve was aligned to the center of the image on a SD-OCT imaging device with a mouse retina lens with a 50° field of view (Bioptigen Envisu R2210 VHR, Bioptigen, USA). The eyes were kept moisturized with eye drops (Systance Ultra, Alcon) and an eye gel (Vidisic carbogen, Bausch & Lomb). The following protocols (a-scan × b-scan × frame) for both eyes were run: (1) linear b-scan, 1.0 mm, 1,000 × 2 × 24 (fast fundus); (2) rectangular, 1.8 × 1.8 mm, 1,000 × 100 × 6 × 1 (high-resolution volume); (3) rectangular, 1.8 × 1.8 mm, 400 × 400 × 4 × 1 (square pixel volume); and (4) radial, 1.8 × 1.8 mm, 1,000 × 100 × 6 × 1 (high-resolution radial volume). The frames were averaged on the InVivoVue Reader software (Bioptigen, USA) and analyzed on Diver 3.4.4 software (Bioptigen, USA) for the VIP for the ONL.

Optokinetic head-tracking response

Optokinetic head-tracking response (OKT) was measured as previously described.[11,23,24] The testing was performed in awake and non-restrained mice. Mice were placed on a pedestal surrounded by four displays that create a visual drum for the mice. The grating was set at 12 degrees/s (spatial frequency). The tracking was recorded in a clockwise (CW) or counterclockwise (CCW) direction. The drum rotation was random from trial to trial, and the experimenter made a forced-choice decision between CW and CCW rotation. The maximum spatial frequency capable of driving head tracking was determined first (visual acuity threshold). The contrast sensitivity was measured at 0.032, 0.064, 0.092, 0.103, 0.192, and 0.272 cycles/degree (spatial frequency). The recording was done twice per mouse. Mice were measured at 1, 3, 6, 9, and 12 months of age at random for the blinded experimenter. The eyes of mice measured in DL-AAA, PBS, and rAAV-h*CRB* injection experiments were recorded separately (treated versus nontreated eye).

Morphological and immunohistochemical analysis

Eyes were collected at the time points of 1, 3, 6, 9, and 12 months of age. Mice injected with DL-AAA, PBS, and/or rAAV-*CRB* were collected at 3 months of age. Mouse eyes were compared to (age-matched) littermates or the nontreated eye of the same animal. The eyes were marked on the superior side with a dye for superior-inferior orientation.[60] For morphological analysis, eyes were enucleated and

fixed at room temperature with 4% paraformaldehyde in PBS for 25 min. Then, the eyes were dehydrated for 30 min in 30%, 50%, 70%, 2× 90%, and 2× 100% ethanol, 50:50 ethanol/Technovit 7100 (Kulzer, Wehrheim, Germany), and finally in 100% Technovit 7100 overnight at 4°C.[60] The eyes were sectioned (2 μm), stained with 1% toluidine blue, and mounted with Entellan. The sections were imaged on ×51 native resolution (bright field) on a slide scanner (3DHISTECH Pannoramic 250). For immunohistochemistry, we dehydrated the eyes for 30 min in 15% sucrose in PBS, followed by 30% sucrose in PBS (30 min). A detailed immunohistochemistry protocol is described in Buck et al.[61] Cryosections (7 μm) were rehydrated in PBS, blocked (1 h), stained with the primary antibody overnight at 4°C, washed in PBS three times (10 min), stained with the secondary antibody (Alexa Fluor 488, Alexa Fluor 555, Alexa Fluor 647, Cy3, or Cy5 for 1 h), washed three times in PBS (10 min), and mounted with Vectashield HardSet mounting medium containing DAPI (Vector Laboratories). A Leica TCS SP8 confocal microscope was used for image acquisition. Image analysis was done in Leica X, Fiji ImageJ, and Adobe Photoshop CC2018.

Antibodies

The following primary antibodies were used: glutamine synthetase (1:250; BD Biosciences), rhodopsin (1:500; Millipore), cone arrestin (1:500; Millipore), PKCα (1:250; BD Biosciences), MPP4 AK4 (1:200; homemade[34]), CRB1 AK2 (pH 1.5) (1:200; homemade[8]), CRB2 (1:200[8]), p120-catenin (1:100; BD Biosciences), GFAP (1:200; Dako), CD11b (1:100; BD Biosciences); PLVAP (1:200; BD Pharmingen), and VE-cadherin (1:100; BD Biosciences).

DL-AAA

DL-AAA preparations were prepared on the day of injections. DL-AAA in PBS was dissolved/deprotonated by dropwise addition 10 M NaOH. Then, the pH was raised to 7.3 by adding hydrochloric acid (37% [w/v] fuming) dropwise. The volume was adjusted with PBS to 100, 150, or 200 μg/μL. The final solution was filter-sterilized (0.022 μm). A volume of 1 μL of DL-AAA or PBS was injected intravitreally in one eye corresponding to 0 (PBS), 100, 150, or 200 μg of DL-AAA.

Generation and purification of rAAV vectors

The pAAV2-AmpR-inverted terminal repeat (ITR)-CMVmin-h*CRB1*co-spA-ITR or pAAV-AmpR-ITR-CMV-h*CRB2*co-SpA-ITR plasmids consist of the flanking ITRs of AAV2, the minimal CMV promoter (for h*CRB1* expression), the full-length CMV promoter (for h*CRB2* expression), the human codon-optimized *CRB1* or *CRB2* cDNA, and a 48-bp synthetic polyadenylation (spA) sequence.[13] The plasmid DNA was produced in Sure-2 cells and extracted on a anion exchange column (endotoxin range, 1–10 endotoxin units [EU]/μg). Endotoxin levels were not measured, and potential endotoxins were not removed from plasmid preps. The hCRB1 and hCRB2 coding sequences used in our rAAV-vectors are highly similar to the mCRB1 or mCRB2 coding sequence proteins. hCRB1/mCRB1 (1,406 and 1,405 aa; UniProtKB: P82279 and Q8VHS2, respectively) and hCRB2/mCRB2 (1,285 and 1282 aa; UniProtKB: Q5IJ48 and Q80YA8, respectively) proteins expressed at the OLM are similar in size and have similar domains (signal peptide, EGF-like, LamG, transmembrane, FERM, PDZ ERLI).[9,14] The pAAV-h*CRB* plasmid, pHelper, and pXX2-ShH10F were co-transfected in 10× 15-cm dishes of 80% confluent HEK293T cells to generate rAAV2/ShH10^{Y445F}.CMV.h*CRB2*co.spA or rAAV2/ShH10^{Y445F}.CMVmin.h*CRB1*co.spA viral particles. After benzonase treatment, the lysates were ultracentrifuged onto an iodixanol density gradient. The purified rAAV stock was filter-sterilized and then concentrated on an amplicon spin column (100,000 nominal molecular weight limit [NMWL]). All viral titers were determined by quantitative PCR. The final rAAV preparation was stored in 0.001% Pluronic F-68 in PBS at −80°C. No major contaminants were found for the rAAV-ShH10^{Y445F}.CMVmin.h*CRB1*co when a 10^{10} viral genome (vg) rAAV sample was denatured by lithium dodecyl sulfate (LDS) sample buffer, dithiothreitol (DTT; reducing agent), and heat (96°C; 5 min) and loaded on a SDS-PAGE gel (NuPAGE Bis-Tris mini gel) and protein-stained (Pierce silver stain kit; see Figure S5T).

rAAV and DL-AAA injection

Mice were anesthetized with 100 mg/kg ketamine and 5 mg/mL xylazine intraperitoneally, and the pupils were dilated using atropine drops (5 mg/mL). The pain was blocked locally by topically applying lidocaine (10 mg/mL) on a cotton swap to the eye and surrounding tissue. rAAV-h*CRB1* or rAAV-h*CRB2* was injected intravitreally in one eye (around 50/50 right versus left eye) at P21-old mice (1 μL; 10^{10} vg). AAA-DL or PBS was injected intravitreally in one eye in 2-month-old mice (1 μL). A Hamilton 10-μL needle was used for AAA, PBS, and rAAV-h*CRB* injections. The injected and noninjected eyes were washed with hypromellose (3 mg/mL) drops, covered with chloramphenicol (Teva, 10 mg/g), and placed on a heating mat for recovery.

Quantification and measurement for spidergrams

The thickness of the retina (ILM to OLM) was measured on plastic sections at 0.5, 1.0, 1.5, and 2.0 mm distance to the ONH, as previously described.[16,60] Three measurements on three different sections were averaged per mouse. Bright-field images were taken on a slide scanner (3DHISTECH Pannoramic 250) at ×51 native resolution.

Normalized CRB2 protein and p120-catenin quantification and OLM breaks

We stained against nuclei (DAPI), CRB2 (second antibody: anti-rabbit Cy3) and p120-catenin (second antibody: anti-mouse Alexa Fluor 488) using a master mix at the same time with one *Crb1*^{KO}*Crb2*$^{Flox/WT}$ and one *Crb1*^{KO}*Crb2*^{LowMGC} eye on each glass slide (total of six slides; total animals: n = 6 *Crb1*^{KO} mice, n = 6 *Crb1*^{KO}*Crb2*^{LowMGC} mice). Three sections per eye were imaged with the same laser and gain settings (12 images per mouse; >1 mm retinal length analyzed for periphery and central area). Two images in the periphery and two images in the central area (inferior and superior; four images per section) were made. Confocal microscopy was done in one run (total of 144 images, 12 animals). An additional group of *Crb2*$^{Flox/Flox}$, *Crb2*$^{\Delta Rods}$, *Crb1*^{KO}*Crb2*$^{\Delta Rods}$, *Crb1*^{KO}, and additional *Crb1*^{KO} *Crb2*$^{Flox/WT}$ retinas were similarly analyzed in one

immunohistochemical and microscopy session but normalized to the previous *Crb1*^{KO}*Crb2*$^{Flox/WT}$ fluorescence intensity values. The OLM area was defined as an area of ~2.5 μm above and 2.5 μm below the OLM. The fluorescence intensity was measured on grayscale images. The mean gray intensity per pixel over the OLM area was calculated. One OLM disruption was defined as an area without p120-catenin OLM expression ≥1 photoreceptor nuclei/column (2.5 μm). The OLM disruptions were normalized over 100-μm retinal length. The concomitant values were averaged per section, then averaged per mouse, and then averaged over the genotype group (n = 6 mice per group). The images were blinded for the investigator before analysis.

Statistical analysis

All statistical analyses were performed using GraphPad Prism version 7 (GraphPad). Normality was tested by the Kolmogorov-Smirnov test. We performed the following statistical analyses for group comparisons: a two-way ANOVA with a Bonferroni post hoc test (averaging right and left eye of a mouse; Figure 1B–1J; Figures S2 and S3), a two-way ANOVA/mixed model with matched values across columns test with a Bonferroni post hoc test (comparing the treated eye versus the control eye within the same mouse; Figure S5), a one-way ANOVA (Kruskal-Wallis) with a Bonferroni post hoc test (Figure S4), an unpaired t test (Figures 1L, 1M, 2, 3, and 5), or a paired t test (comparing the treated eye versus the control eye within the same mouse; Figure 6).[62] All values are expressed as mean ± SEM unless otherwise indicated. Statistical significant values are defined as follows: *$p < 0.05$, **$p < 0.01$, ***$p < 0.001$.

SUPPLEMENTAL INFORMATION

Supplemental Information can be found online at https://doi.org/10.1016/j.omtm.2020.12.012.

ACKNOWLEDGMENTS

The authors thank Ilse Voshart, Ariadna Rocha Sierra, Martijn Koomen, Charlotte A. Andriessen, and Annelies Boonzaier-van der Laan for technical assistance, and all Wijnholds Lab members for advice on the manuscript. This work was supported by the Foundation Fighting Blindness (TA-GT-0715-0665-LUMC, to J.W.), the Netherlands Organisation for Health Research and Development (ZonMw grant 43200004, to J.W.), and by the Dutch blindness funds (Uitzicht 2013-13, to J.W. and Uitzicht 2018-6, to C.H.A. and J.W.): Rotterdamse Stichting Blindenbelangen, MaculaFonds, Stichting Blindenhulp, Landelijke Stichting voor Blinden en Slechtzienden, Algemene Nederlandse Vereniging ter Voorkoming van Blindheid, and Stichting Blinden-Penning.

AUTHOR CONTRIBUTIONS

Conceptualization, T.M.B. and J.W.; methodology, T.M.B and J.W.; investigation, T.M.B. and R.M.V.; formal analysis, T.M.B. and J.W.; writing – original draft, T.M.B.; writing – review & editing, T.M.B., C.H.A., and J.W.; funding acquisition, C.H.A. and J.W.; resources, R.M.V. and J.W.; supervision, J.W.

DECLARATION OF INTERESTS

The authors declare no competing interests. The LUMC is the holder of patent number PCT/NL2014/050549, which describes the potential clinical use of CRB2. J.W. is listed as co-inventor of this patent and is an employee of the LUMC.

REFERENCES

1. Talib, M., van Schooneveld, M.J., van Genderen, M.M., Wijnholds, J., Florijn, R.J., Ten Brink, J.B., Schalij-Delfos, N.E., Dagnelie, G., Cremers, F.P.M., Wolterbeek, R., et al. (2017). Genotypic and phenotypic characteristics of *CRB1*-associated retinal dystrophies: a long-term follow-up study. Ophthalmology *124*, 884–895.
2. Vincent, A., Ng, J., Gerth-Kahlert, C., Tavares, E., Maynes, J.T., Wright, T., Tiwari, A., Tumber, A., Li, S., Hanson, J.V.M., et al. (2016). Biallelic mutations in *CRB1* underlie autosomal recessive familial foveal retinoschisis. Invest. Ophthalmol. Vis. Sci. *57*, 2637–2646.
3. Tsang, S.H., Burke, T., Oll, M., Yzer, S., Lee, W., Xie, Y.A., and Allikmets, R. (2014). Whole exome sequencing identifies *CRB1* defect in an unusual maculopathy phenotype. Ophthalmology *121*, 1773–1782.
4. Quinn, P.M., Buck, T.M., Mulder, A.A., Ohonin, C., Alves, C.H., Vos, R.M., Bialecka, M., van Herwaarden, T., van Dijk, E.H.C., Talib, M., et al. (2019). Human iPSC-derived retinas recapitulate the fetal CRB1 CRB2 complex formation and demonstrate that photoreceptors and müller glia are targets of AAV5. Stem Cell Reports *12*, 906–919.
5. Quinn, P.M., Mulder, A.A., Henrique Alves, C., Desrosiers, M., de Vries, S.I., Klooster, J., Dalkara, D., Koster, A.J., Jost, C.R., and Wijnholds, J. (2019). Loss of CRB2 in Müller glial cells modifies a *CRB1*-associated retinitis pigmentosa phenotype into a Leber congenital amaurosis phenotype. Hum. Mol. Genet. *28*, 105–123.
6. Pellissier, L.P., Lundvig, D.M.S., Tanimoto, N., Klooster, J., Vos, R.M., Richard, F., Sothilingam, V., Garcia Garrido, M., Le Bivic, A., Seeliger, M.W., and Wijnholds, J. (2014). CRB2 acts as a modifying factor of *CRB1*-related retinal dystrophies in mice. Hum. Mol. Genet. *23*, 3759–3771.
7. van Rossum, A.G.S.H., Aartsen, W.M., Meuleman, J., Klooster, J., Malysheva, A., Versteeg, I., Arsanto, J.-P.P., Le Bivic, A., and Wijnholds, J. (2006). Pals1/Mpp5 is required for correct localization of Crb1 at the subapical region in polarized Muller glia cells. Hum. Mol. Genet. *15*, 2659–2672.
8. van de Pavert, S.A., Kantardzhieva, A., Malysheva, A., Meuleman, J., Versteeg, I., Levelt, C., Klooster, J., Geiger, S., Seeliger, M.W., Rashbass, P., et al. (2004). Crumbs homologue 1 is required for maintenance of photoreceptor cell polarization and adhesion during light exposure. J. Cell Sci. *117*, 4169–4177.
9. Quinn, P.M., Pellissier, L.P., and Wijnholds, J. (2017). The CRB1 complex: following the trail of Crumbs to a feasible gene therapy strategy. Front. Neurosci. *11*, 175.
10. van de Pavert, S.A., Meuleman, J., Malysheva, A., Aartsen, W.M., Versteeg, I., Tonagel, F., Kamphuis, W., McCabe, C.J., Seeliger, M.W., and Wijnholds, J. (2007). A single amino acid substitution (Cys249Trp) in Crb1 causes retinal degeneration and deregulates expression of pituitary tumor transforming gene *Pttg1*. J. Neurosci. *27*, 564–573.
11. Alves, C.H., Boon, N., Mulder, A.A., Koster, A.J., Jost, C.R., and Wijnholds, J. (2019). CRB2 loss in rod photoreceptors is associated with progressive loss of retinal contrast sensitivity. Int. J. Mol. Sci. *20*, 4069.
12. van de Pavert, S.A., Sanz, A.S., Aartsen, W.M., Vos, R.M., Versteeg, I., Beck, S.C., Klooster, J., Seeliger, M.W., and Wijnholds, J. (2007). Crb1 is a determinant of retinal apical Müller glia cell features. Glia *55*, 1486–1497.
13. Pellissier, L.P., Quinn, P.M., Alves, C.H., Vos, R.M., Klooster, J., Flannery, J.G., Heimel, J.A., and Wijnholds, J. (2015). Gene therapy into photoreceptors and Müller glial cells restores retinal structure and function in *CRB1* retinitis pigmentosa mouse models. Hum. Mol. Genet. *24*, 3104–3118.
14. Boon, N., Wijnholds, J., and Pellissier, L.P. (2020). Research models and gene augmentation therapy for *CRB1* retinal dystrophies. Front. Neurosci. *14*, 860.
15. Alves, C.H., Sanz, A.S., Park, B., Pellissier, L.P., Tanimoto, N., Beck, S.C., Huber, G., Murtaza, M., Richard, F., Sridevi Gurubaran, I., et al. (2013). Loss of CRB2 in the mouse retina mimics human retinitis pigmentosa due to mutations in the *CRB1* gene. Hum. Mol. Genet. *22*, 35–50.

16. Quinn, P.M., Alves, C.H., Klooster, J., and Wijnholds, J. (2018). CRB2 in immature photoreceptors determines the superior-inferior symmetry of the developing retina to maintain retinal structure and function. Hum. Mol. Genet. *27*, 3137–3153.

17. West, E.L., Pearson, R.A., Tschernutter, M., Sowden, J.C., MacLaren, R.E., and Ali, R.R. (2008). Pharmacological disruption of the outer limiting membrane leads to increased retinal integration of transplanted photoreceptor precursors. Exp. Eye Res. *86*, 601–611.

18. Kato, S., Ishita, S., Sugawara, K., and Mawatari, K. (1993). Cystine/glutamate antiporter expression in retinal Müller glial cells: implications for DL-alpha-aminoadipate toxicity. Neuroscience *57*, 473–482.

19. Yu, W.Q., Eom, Y.S., Shin, J.A., Nair, D., Grzywacz, S.X.Z., Grzywacz, N.M., Craft, C.M., and Lee, E.J. (2016). Reshaping the cone-mosaic in a rat model of retinitis pigmentosa: modulatory role of ZO-1 expression in DL-alpha-aminoadipic acid reshaping. PLoS ONE *11*, e0151668.

20. Shen, W., Zhang, J., Chung, S.H., Hu, Y., Ma, Z., and Gillies, M.C. (2011). Submacular DL-α-aminoadipic acid eradicates primate photoreceptors but does not affect luteal pigment or the retinal vasculature. Invest. Ophthalmol. Vis. Sci. *52*, 119–127.

21. Jimeno, D., Feiner, L., Lillo, C., Teofilo, K., Goldstein, L.S.B., Pierce, E.A., and Williams, D.S. (2006). Analysis of kinesin-2 function in photoreceptor cells using synchronous *Cre*-loxP knockout of *Kif3a* with *RHO-Cre*. Invest. Ophthalmol. Vis. Sci. *47*, 5039–5046.

22. Murray, S.A., Eppig, J.T., Smedley, D., Simpson, E.M., and Rosenthal, N. (2012). Beyond knockouts: cre resources for conditional mutagenesis. Mamm. Genome *23*, 587–599.

23. Prusky, G.T., West, P.W.R., and Douglas, R.M. (2000). Behavioral assessment of visual acuity in mice and rats. Vision Res. *40*, 2201–2209.

24. Prusky, G.T., Alam, N.M., Beekman, S., and Douglas, R.M. (2004). Rapid quantification of adult and developing mouse spatial vision using a virtual optomotor system. Invest. Ophthalmol. Vis. Sci. *45*, 4611–4616.

25. Prusky, G.T., and Douglas, R.M. (2004). Characterization of mouse cortical spatial vision. Vision Res. *44*, 3411–3418.

26. de Souza, C.F., Nivison-Smith, L., Christie, D.L., Polkinghorne, P., McGhee, C., Kalloniatis, M., and Acosta, M.L. (2016). Macromolecular markers in normal human retina and applications to human retinal disease. Exp. Eye Res. *150*, 135–148.

27. Krizaj, D., Demarco, S.J., Johnson, J., Strehler, E.E., and Copenhagen, D.R. (2002). Cell-specific expression of plasma membrane calcium ATPase isoforms in retinal neurons. J. Comp. Neurol. *451*, 1–21.

28. Pellissier, L.P., Alves, C.H., Quinn, P.M., Vos, R.M., Tanimoto, N., Lundvig, D.M.S., Dudok, J.J., Hooibrink, B., Richard, F., Beck, S.C., et al. (2013). Targeted ablation of *Crb1* and *Crb2* in retinal progenitor cells mimics Leber congenital amaurosis. PLoS Genet. *9*, e1003976.

29. Bazellières, E., Aksenova, V., Barthélémy-Requin, M., Massey-Harroche, D., and Le Bivic, A. (2018). Role of the Crumbs proteins in ciliogenesis, cell migration and actin organization. Semin. Cell Dev. Biol. *81*, 13–20.

30. Bulgakova, N.A., and Knust, E. (2009). The Crumbs complex: from epithelial-cell polarity to retinal degeneration. J. Cell Sci. *122*, 2587–2596.

31. Margolis, B. (2018). The Crumbs3 polarity protein. Cold Spring Harb. Perspect. Biol. *10*, a027961.

32. Assémat, E., Crost, E., Ponserre, M., Wijnholds, J., Le Bivic, A., and Massey-Harroche, D. (2013). The multi-PDZ domain protein-1 (MUPP-1) expression regulates cellular levels of the PALS-1/PATJ polarity complex. Exp. Cell Res. *319*, 2514–2525.

33. Alves, C.H., Pellissier, L.P., Vos, R.M., Garcia Garrido, M., Sothilingam, V., Seide, C., Beck, S.C., Klooster, J., Furukawa, T., Flannery, J.G., et al. (2014). Targeted ablation of *Crb2* in photoreceptor cells induces retinitis pigmentosa. Hum. Mol. Genet. *23*, 3384–3401.

34. Kantardzhieva, A., Gosens, I., Alexeeva, S., Punte, I.M., Versteeg, I., Krieger, E., Neefjes-Mol, C.A., den Hollander, A.I., Letteboer, S.J.F.F., Klooster, J., et al. (2005). MPP5 recruits MPP4 to the CRB1 complex in photoreceptors. Invest. Ophthalmol. Vis. Sci. *46*, 2192–2201.

35. Pearson, R.A., Barber, A.C., West, E.L., MacLaren, R.E., Duran, Y., Bainbridge, J.W., Sowden, J.C., and Ali, R.R. (2010). Targeted disruption of outer limiting membrane junctional proteins (Crb1 and ZO-1) increases integration of transplanted photoreceptor precursors into the adult wild-type and degenerating retina. Cell Transplant. *19*, 487–503.

36. Pellissier, L.P., Hoek, R.M., Vos, R.M., Aartsen, W.M., Klimczak, R.R., Hoyng, S.A., Flannery, J.G., and Wijnholds, J. (2014). Specific tools for targeting and expression in Müller glial cells. Mol. Ther. Methods Clin. Dev. *1*, 14009.

37. Klimczak, R.R., Koerber, J.T., Dalkara, D., Flannery, J.G., and Schaffer, D.V. (2009). A novel adeno-associated viral variant for efficient and selective intravitreal transduction of rat Müller cells. PLoS ONE *4*, e7467.

38. Chakarova, C.F., Khanna, H., Shah, A.Z., Patil, S.B., Sedmak, T., Murga-Zamalloa, C.A., Papaioannou, M.G., Nagel-Wolfrum, K., Lopez, I., Munro, P., et al. (2011). TOPORS, implicated in retinal degeneration, is a cilia-centrosomal protein. Hum. Mol. Genet. *20*, 975–987.

39. Aartsen, W.M., Arsanto, J.P., Chauvin, J.P., Vos, R.M., Versteeg, I., Cardozo, B.N., Bivic, A.L., and Wijnholds, J. (2009). PSD95β regulates plasma membrane Ca^{2+} pump localization at the photoreceptor synapse. Mol. Cell. Neurosci. *41*, 156–165.

40. Klaassen, I., Van Noorden, C.J.F., and Schlingemann, R.O. (2013). Molecular basis of the inner blood-retinal barrier and its breakdown in diabetic macular edema and other pathological conditions. Prog. Retin. Eye Res. *34*, 19–48.

41. Wisniewska-Kruk, J., van der Wijk, A.E., van Veen, H.A., Gorgels, T.G.M.F., Vogels, I.M.C., Versteeg, D., Van Noorden, C.J.F., Schlingemann, R.O., and Klaassen, I. (2016). Plasmalemma vesicle-associated protein has a key role in blood-retinal barrier loss. Am. J. Pathol. *186*, 1044–1054.

42. Timmers, A.M., Newmark, J.A., Turunen, H.T., Farivar, T., Liu, J., Song, C., Ye, G.J., Pennock, S., Gaskin, C., Knop, D.R., and Shearman, M.S. (2020). Ocular inflammatory response to intravitreal injection of adeno-associated virus vector: relative contribution of genome and capsid. Hum. Gene Ther. *31*, 80–89.

43. Newman, E., and Reichenbach, A. (1996). The Müller cell: a functional element of the retina. Trends Neurosci. *19*, 307–312.

44. Franze, K., Grosche, J., Skatchkov, S.N., Schinkinger, S., Foja, C., Schild, D., Uckermann, O., Travis, K., Reichenbach, A., and Guck, J. (2007). Muller cells are living optical fibers in the vertebrate retina. Proc. Natl. Acad. Sci. USA *104*, 8287–8292.

45. Newman, E.A. (2015). Glial cell regulation of neuronal activity and blood flow in the retina by release of gliotransmitters. Philos. Trans. R. Soc. Lond. B Biol. Sci. *370*, 1–9.

46. Witmer, A.N., Blaauwgeers, H.G., Weich, H.A., Alitalo, K., Vrensen, G.F., and Schlingemann, R.O. (2002). Altered expression patterns of VEGF receptors in human diabetic retina and in experimental VEGF-induced retinopathy in monkey. Invest. Ophthalmol. Vis. Sci. *43*, 849–857.

47. Son, S., Cho, M., and Lee, J. (2020). Crumbs proteins regulate layered retinal vascular development required for vision. Biochem. Biophys. Res. Commun. *521*, 939–946.

48. Kim, J.H., Kim, J.H., Yu, Y.S., Kim, D.H., and Kim, K.-W. (2009). Recruitment of pericytes and astrocytes is closely related to the formation of tight junction in developing retinal vessels. J. Neurosci. Res. *87*, 653–659.

49. Gardner, T.W., Lieth, E., Khin, S.A., Barber, A.J., Bonsall, D.J., Lesher, T., Rice, K., and Brennan, W.A., Jr. (1997). Astrocytes increase barrier properties and ZO-1 expression in retinal vascular endothelial cells. Invest. Ophthalmol. Vis. Sci. *38*, 2423–2427.

50. Witmer, A.N., Vrensen, G.F.J.M., Van Noorden, C.J.F., and Schlingemann, R.O. (2003). Vascular endothelial growth factors and angiogenesis in eye disease. Prog. Retin. Eye Res. *22*, 1–29.

51. Meeson, A.P., Argilla, M., Ko, K., Witte, L., and Lang, R.A. (1999). VEGF deprivation-induced apoptosis is a component of programmed capillary regression. Development *126*, 1407–1415.

52. Koerber, J.T., Klimczak, R., Jang, J.-H.H., Dalkara, D., Flannery, J.G., and Schaffer, D.V. (2009). Molecular evolution of adeno-associated virus for enhanced glial gene delivery. Mol. Ther. *17*, 2088–2095.

53. Xiao, Z., Patrakka, J., Nukui, M., Chi, L., Niu, D., Betsholtz, C., Pikkarainen, T., Vainio, S., and Tryggvason, K. (2011). Deficiency in Crumbs homolog 2 (*Crb2*) affects gastrulation and results in embryonic lethality in mice. Dev. Dyn. *240*, 2646–2656.

54. van den Hurk, J.A., Rashbass, P., Roepman, R., Davis, J., Voesenek, K.E., Arends, M.L., Zonneveld, M.N., van Roekel, M.H., Cameron, K., Rohrschneider, K., et al. (2005). Characterization of the *Crumbs homolog 2* (*CRB2*) gene and analysis of its role in retinitis pigmentosa and Leber congenital amaurosis. Mol. Vis. *11*, 263–273.

55. Dolón, J.F., Paniagua, A.E., Valle, V., Segurado, A., Arévalo, R., Velasco, A., and Lillo, C. (2018). Expression and localization of the polarity protein CRB2 in adult mouse brain: a comparison with the CRB1^{rd8} mutant mouse model. Sci. Rep. *8*, 11652.

56. Zhao, M., Andrieu-Soler, C., Kowalczuk, L., Paz Cortés, M., Berdugo, M., Dernigoghossian, M., Halili, F., Jeanny, J.C., Goldenberg, B., Savoldelli, M., et al. (2015). A new CRB1 rat mutation links Müller glial cells to retinal telangiectasia. J. Neurosci. *35*, 6093–6106.

57. Shen, W., Li, S., Chung, S.H., and Gillies, M.C. (2010). Retinal vascular changes after glial disruption in rats. J. Neurosci. Res. *88*, 1485–1499.

58. Li, Y., Busoy, J.M., Zaman, B.A.A., Tan, Q.S.W., Tan, G.S.W., Barathi, V.A., Cheung, N., Wei, J.J.Y., Hunziker, W., Hong, W., et al. (2018). A novel model of persistent retinal neovascularization for the development of sustained anti-VEGF therapies. Exp. Eye Res. *174*, 98–106.

59. Roesch, K., Jadhav, A.P., Trimarchi, J.M., Stadler, M.B., Roska, B., Sun, B.B., and Cepko, C.L. (2008). The transcriptome of retinal Müller glial cells. J. Comp. Neurol. *509*, 225–238.

60. Alves, C.H., and Wijnholds, J. (2018). AAV gene augmentation therapy for CRB1-associated retinitis pigmentosa. In Methods in Molecular Biology, C.J.F. Boon and J. Wijnholds, eds. (Humana Press), pp. 135–151.

61. Buck, T.M., Pellissier, L.P., Vos, R.M., van Dijk, E.H.C.C., Boon, C.J.F., and Wijnholds, J. (2018). AAV serotype testing on cultured human donor retinal explants. In Methods in Molecular Biology, C.J.F. Boon and J. Wijnholds, eds. (Humana Press), pp. 275–288.

62. Heynen, S.R., Meneau, I., Caprara, C., Samardzija, M., Imsand, C., Levine, E.M., and Grimm, C. (2013). CDC42 is required for tissue lamination and cell survival in the mouse retina. PLoS ONE *8*, e53806.

OMTM, Volume *20*

Supplemental Information

AAV-*CRB2* protects against vision loss in an inducible *CRB1* retinitis pigmentosa mouse model

Thilo M. Buck, Rogier M. Vos, C. Henrique Alves, and Jan Wijnholds

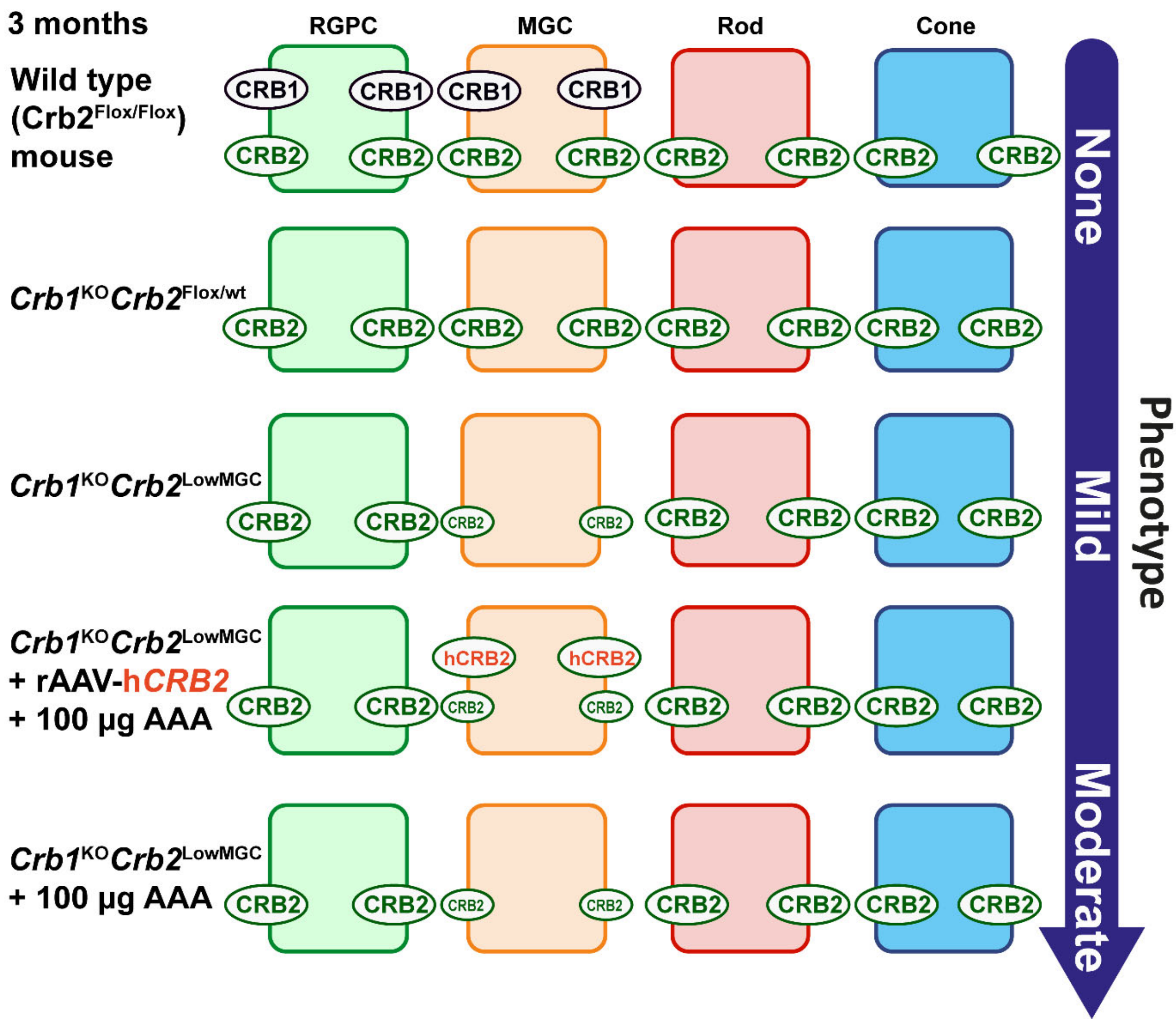

Figure S1. Graphical schematic representation of CRB localization and the severity of the phenotype in 3-month-old mice. Graphical schematic representation of CRB1 and CRB2 in radial glial progenitor cells (RGPC), Müller glial cells (MGC), rod and cone photoreceptors in wildtype ($Crb2^{\text{Flox/Flox}}$), $Crb1^{\text{KO}}Crb2^{\text{Flox/Flox}}$ (Control to $Crb1^{\text{KO}}Crb2^{\text{LowMGC}}$), $Crb1^{\text{KO}}Crb2^{\text{LowMGC}}$, $Crb1^{\text{KO}}Crb2^{\text{LowMGC}}$ + 100 µg DL-AAA intravitreally (i.vit) injected at 2 months (Control to rAAV-*CRB2* injected mice), and $Crb1^{\text{KO}}Crb2^{\text{LowMGC}}$ + rAAV-h*CRB2* i.vit injected at postnatal day 21 + 100 µg DL-AAA i.vit injected at 2 months. rAAV, recombinant adeno-associated viral vector; i.vit., intravitreal. Modified from [5].

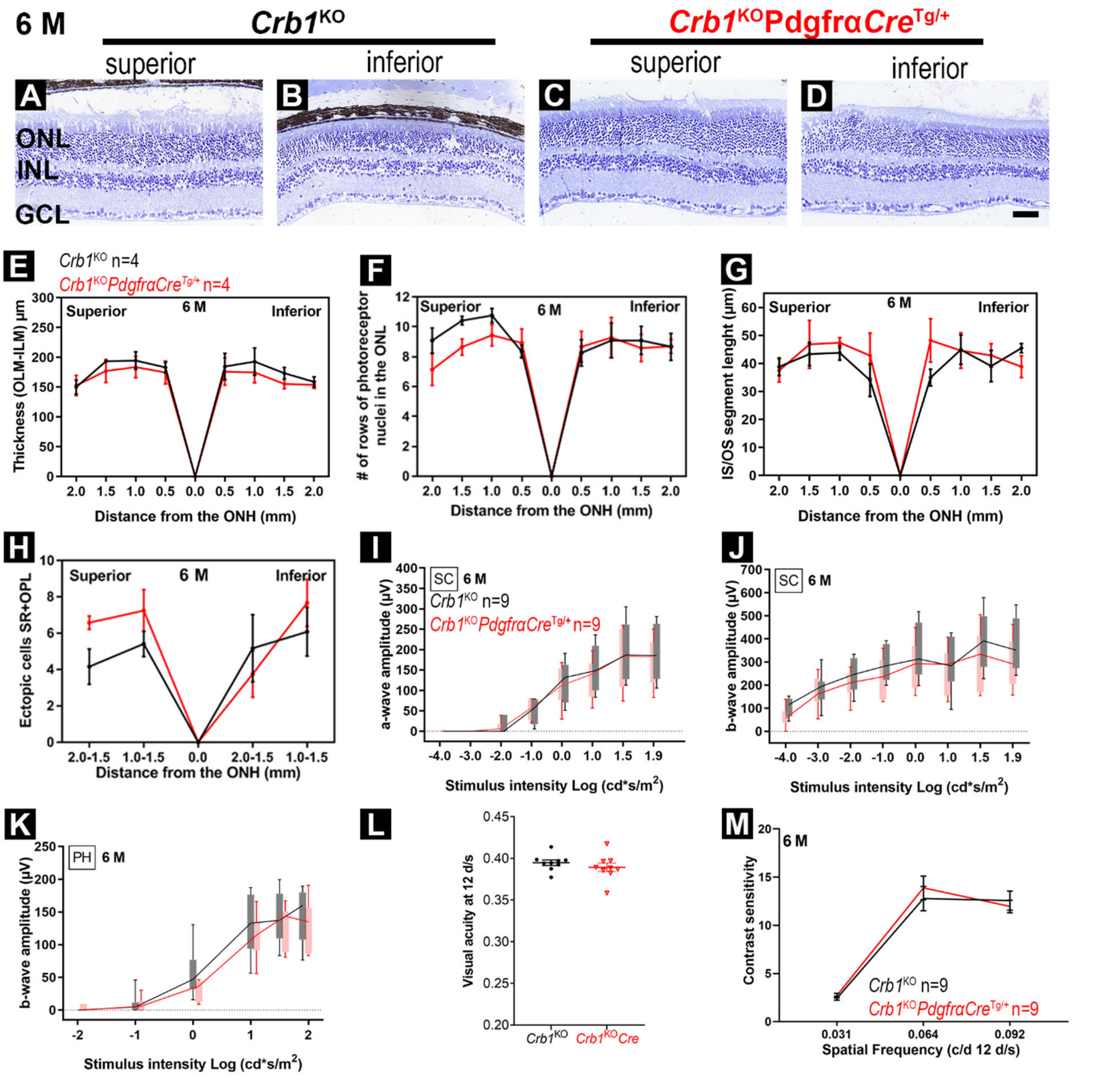

Figure S2. Cre-expression in Müller glial cells does not affect retinal morphology, ERG responses, and OKT responses. Toluidine-stained light microscopy of retinal sections from control (*Crb1*^{KO}; A-B) and *Crb1*$^{-/-}$ Pdgfrα*Cre*$^{Tg/+}$ (C-D) mice at 6-month-of-age. Spidergrams for (E) Retinal thickness, (F) number of photoreceptors per row, (G) inner/outer segments of photoreceptor length, and (H) ectopic cells in the subretinal space and outer plexiform layer. Quantitative evaluation of the scotopic single-flash intensity series of the a-wave (I), b-wave (J), and photopic b-wave (K) amplitudes in 6-month old mice. Boxes indicate the 25 and 75% quantile range, whiskers indicate the 5 and 95% quantiles, and the intersection of line and error bar indicates the median of the data (box-and-whisker plot). (L-M) Optokinetic head tracking response at 6-month old mice. (L) Spatial frequency threshold (visual acuity). (M) Contrast sensitivity threshold at different spatial frequencies. *P<0.05; **p<0.01, ***P<0.001.

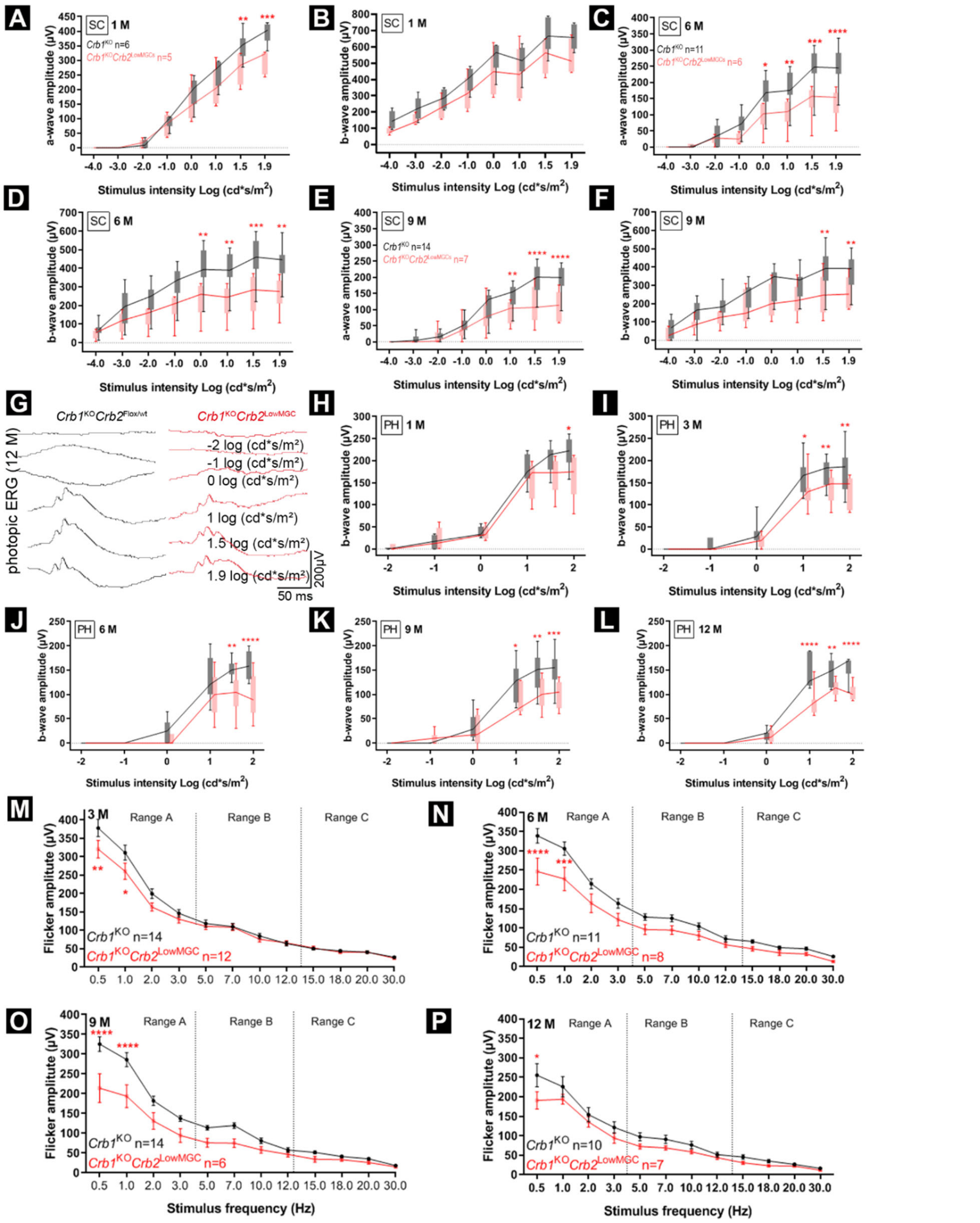

Figure S3. Decreased retinal function (ERG) and vision-guided (OKT) behavior in $Crb1^{KO}Crb2^{LowMGC}$ compared to $Crb1^{KO}Crb2^{Flox/wt}$ age-matched littermates. $Crb1^{KO}Crb2^{LowMGC}$ measurements are indicated in red (experimental group) and $Crb1^{KO}Crb2^{Flox/wt}$ age-matched littermates in black (control group). Electroretinographic analysis of the retinal function: (A-F) Scotopic [SC] single-flash intensity series ERG from representative animals at 1-, 6-, 9-month old mice of age. (G-L) Photopic [PH] single-flash ERG at different light intensities (-2, -1, 0, 1, 1.5, 1.9 log cd s/m² light intensity at 30 cd/m² background light): (G) Photopic ERG traces of representative 12-month-

old animals. (H-L) Photopic b-wave amplitudes at 1-, 3-, 6-, 9-, and 12-month-old mice (H-L). No statistical analysis was performed for (A-L). (M-P) Time course of flicker response amplitudes from 3-, 6-, 9-, and 12-month-old mice (Mean±SEM; M-P; *P<0.05; **p<0.01, ***P<0.001). Animal numbers (control vs. experimental): 1-month (n=6; n=6), 3-month (n=14; n=12), 6-month (n=11; n=6), 9-month (n=12; n=7), 12-month (n=7; n=8).

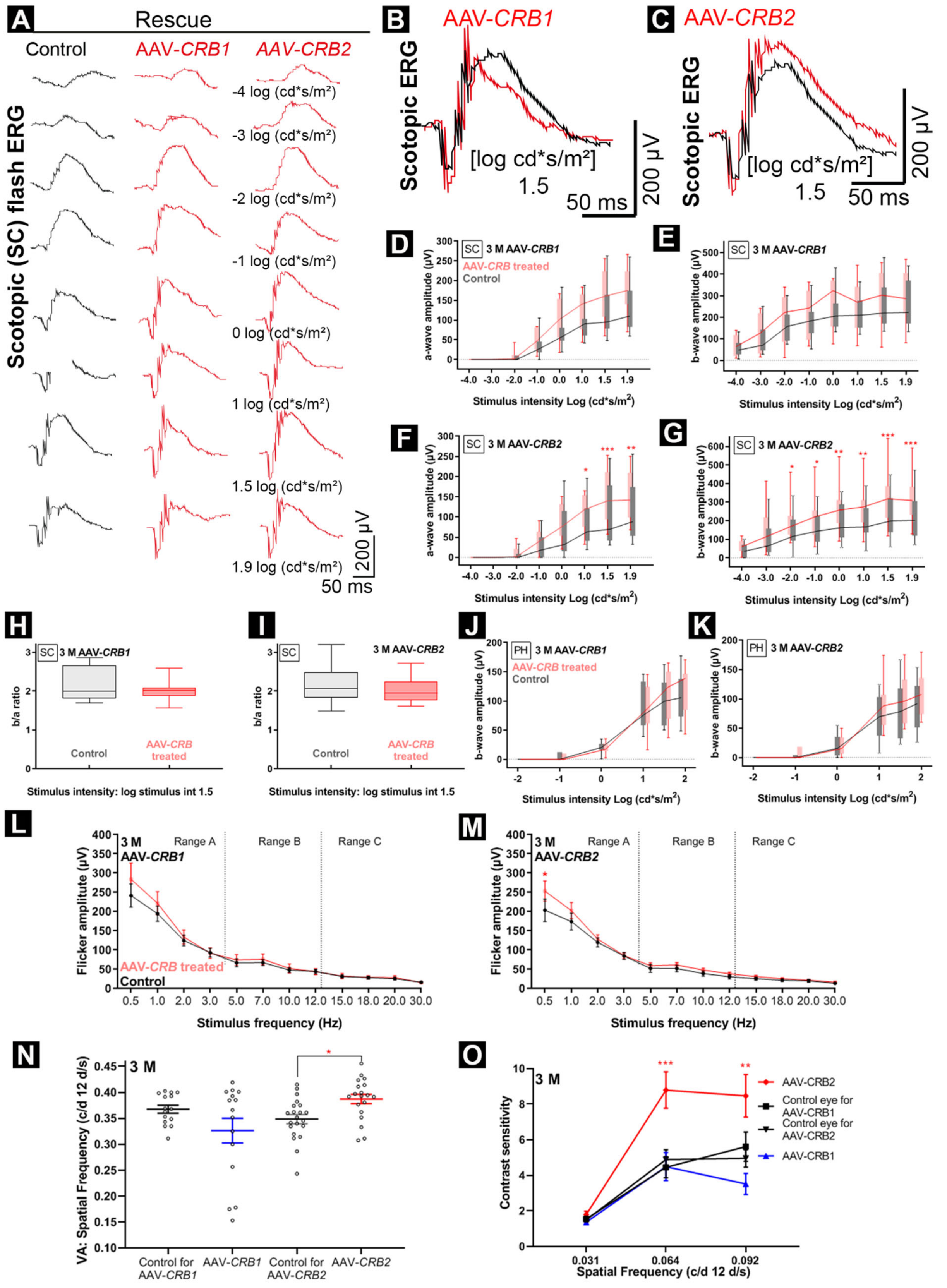

Figure S4. Retinal function and visual-guided head tracking in rAAV-h*CRB* treated *Crb1*^{KO}*Crb2*^{LowMGC} compared to control eyes. Both eyes received a 100 µg DL-AAA treatment at 2-months. Eyes were measured at three months. The ERG traces of the rAAV treated eye (rAAV-h*CRB* injected at postnatal day 21) are indicated in

red (experimental group) and compared to the control eye (Not treated) not receiving the AAV therapy (black trace, control group). Electroretinographic analysis of the retinal function: (A-C) Scotopic [SC] single-flash intensity series (-4, -3 , -2, -1, 0, 1, 1.5, 1.9 log cd s/m² light intensity) ERG from representative animals in 3-month old mice. (B-C) Superimposed scotopic [SC] single-flash ERG traces at 1.5 log cd s/m² intensity from representative animals. (D) Scotopic a-wave of rAAV-h*CRB1* injected mice vs. the control eye. (E) Scotopic b-wave of rAAV-h*CRB1* injected mice vs. control eye. (F) Scotopic a-wave of rAAV-h*CRB2* injected mice vs. control eye. (G) Scotopic b-wave of rAAV-h*CRB2* injected mice vs. control eye. (H-I) b-wave/a-wave ratio of AAV-h*CRB1* or AAV-h*CRB2* at single-flash ERG traces at 1.5 log cd s/m² intensity. (J-K) Photopic b-wave amplitudes in 3-month-old mice injected with rAAV-h*CRB1* or rAAV-h*CRB2* vs. control eye. No statistical analysis was performed (A-K). (L-M) Flicker response amplitudes from 3-month-old mice injected with rAAV-h*CRB1* or rAAV-h*CRB2* vs, the control eye. Number of animals (Control vs experimental): rAAV-h*CRB1* (n=16; n=14). rAAV-h*CRB2* (n=18; n=16). (N-P Optokinetic head tracking response: (N) Visual acuity (animals indicates as black circles); contrast sensitivity OKT (Number of animals: rAAV-h*CRB1* n=16; rAAV-h*CRB2*: n=21). Mean±SEM; M-P; *P<0.05; **p<0.01, ***P<0.001.

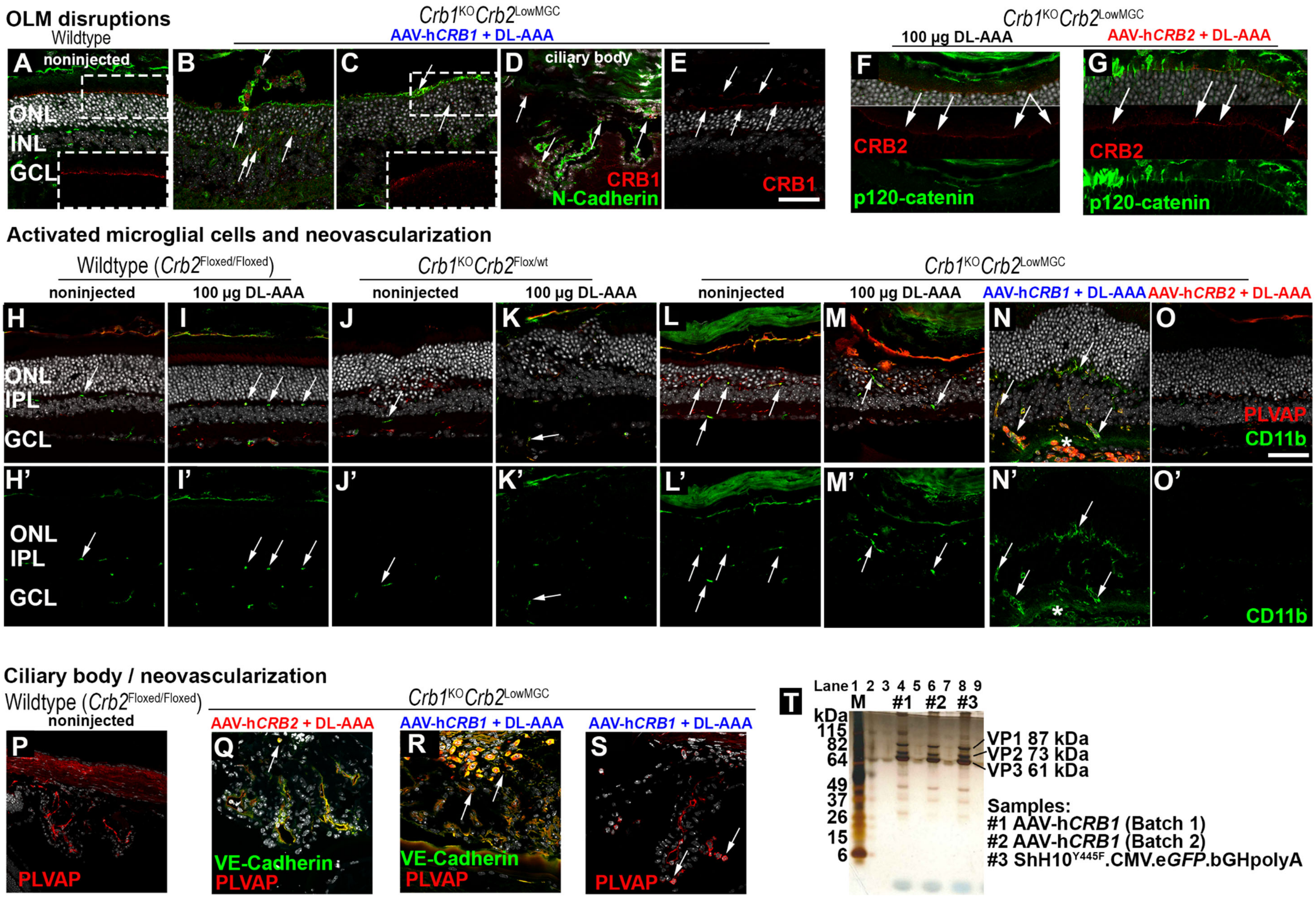

OLM disruptions
Wildtype
Crb1KOCrb2LowMGC
AAV-hCRB1 + DL-AAA
A noninjected
ONL
INL
GCL
B
C
D ciliary body
CRB1
N-Cadherin
E
CRB1
Crb1KOCrb2LowMGC
100 µg DL-AAA
AAV-hCRB2 + DL-AAA
F
CRB2
p120-catenin
G
CRB2
p120-catenin
Activated microglial cells and neovascularization
Wildtype (Crb2Floxed/Floxed)
Crb1KOCrb2Flox/wt
Crb1KOCrb2LowMGC
noninjected
100 µg DL-AAA
noninjected
100 µg DL-AAA
noninjected
100 µg DL-AAA
AAV-hCRB1 + DL-AAA
AAV-hCRB2 + DL-AAA
H I J K L M N O
ONL
IPL
GCL
PLVAP
CD11b
H' I' J' K' L' M' N' O'
ONL
IPL
GCL
CD11b
Ciliary body / neovascularization
Wildtype (Crb2Floxed/Floxed)
noninjected
Crb1KOCrb2LowMGC
AAV-hCRB2 + DL-AAA
AAV-hCRB1 + DL-AAA
AAV-hCRB1 + DL-AAA
P
PLVAP
Q
VE-Cadherin
PLVAP
R
VE-Cadherin
PLVAP
S
PLVAP
T
Lane 1 2 3 4 5 6 7 8 9
M #1 #2 #3
kDa
115
82
64
49
37
26
15
6
VP1 87 kDa
VP2 73 kDa
VP3 61 kDa
Samples:
#1 AAV-hCRB1 (Batch 1)
#2 AAV-hCRB1 (Batch 2)
#3 ShH10Y445F.CMV.eGFP.bGHpolyA

Figure S5. AAV-h*CRB1* increases neovascularization in the ciliary body. Immunohistochemistry of 3-month-old mice. Sections were stained for: (A-E) CRB1 protein (red) at the subapical region and N-cadherin (green) at the adherens junction in (A) wildtype and (B-E) $Crb1^{KO}Crb2^{LowMGC}$ mice injected with rAAV-h*CRB1* and 100 µg DL-AAA (arrows indicate CRB1 protein expression). (F-G) CRB2 protein (red) at the subapical region and p120-catenin (green) at the adherens junction in (F) $Crb1^{KO}Crb2^{LowMGC}$ mice injected with 100 µg DL-AAA and (G) $Crb1^{KO}Crb2^{LowMGC}$ mice injected with 100 µg DL-AAA and rAAV-h*CRB2* (arrows = CRB2 protein expression at OLM). (H-O) PLVAP (red) for (neo-)vascularization and CD11b (green) for microglial activation (arrows). (H'-O') Puncta-like CD11b-positive microglia cells in IPL are dormant and activated CD11b-positive microglia cells in ONL and GCL (arrows). (P-S) The ciliary body of (P) wildtype control mice or $Crb1^{KO}Crb2^{LowMGC}$ mice injected with (Q) rAAV-h*CRB2* or (R-S) rAAV-h*CRB1*. PLVAP (red) and VE-cadherin (green) are vascularization markers. (Neo-)vascularization indicated by arrows in Q-S. (T) $1x10^{10}$ viral genomes (vg) of two batches of $rAAV2/ShH10^{Y445F}$.CMVmin.h*CRB1* vector preparation and one batch of $1x10^{10}$ vg of $rAAV2/ShH10^{Y445F}$.CMV.*GFP* were analysed by silver nitrate staining to detect capsid proteins VP1, VP2, VP3. Note that the silver stained SDS-PAGE gel was overstained (for marker and capsid proteins) to allow evaluation of capsid degradation products and contaminants. Lane 1, protein marker. Lanes 4 and 6, two independent samples of $rAAV2/ShH10^{Y445F}$.h*CRB1* used in this study. Lane 8, a sample of $rAAV2/ShH10^{Y445F}$.*GFP*. Note that lanes 2, 3, 5, 7 and 9 contain overflow from adjacent sample wells. Scale bar=50 µm. Inserts 50 µm height. PLVAP, plasmalemma vesicle associated protein; ONL, outer nuclear layer; INL, inner nuclear layer; GCL, ganglion cell layer. 3-4 eyes per group were analyzed.
